# Major Bleeding and Thromboembolic Events with the On-X Mechanical Aortic Valve Prosthesis: A SWEDEHEART Study

**DOI:** 10.1093/icvts/ivaf182

**Published:** 2025-07-29

**Authors:** Ruixin Lu, Michael Dismorr, Magnus Dalén, Natalie Glaser, Ulrik Sartipy

**Affiliations:** Department of Molecular Medicine and Surgery, Karolinska Institutet, 171 76 Stockholm, Sweden; Department of Molecular Medicine and Surgery, Karolinska Institutet, 171 76 Stockholm, Sweden; Department of Molecular Medicine and Surgery, Karolinska Institutet, 171 76 Stockholm, Sweden; Department of Cardiothoracic Surgery, Karolinska University Hospital, 171 76 Stockholm, Sweden; Department of Molecular Medicine and Surgery, Karolinska Institutet, 171 76 Stockholm, Sweden; Department of Cardiology, Stockholm South General Hospital, 118 83 Stockholm, Sweden; Department of Molecular Medicine and Surgery, Karolinska Institutet, 171 76 Stockholm, Sweden; Department of Cardiothoracic Surgery, Karolinska University Hospital, 171 76 Stockholm, Sweden

**Keywords:** mechanical aortic valve prostheses, On-X aortic valve, major bleeding, thromboembolic events, epidemiology

## Abstract

**Objectives:**

Lifelong anticoagulation therapy is mandatory in patients with mechanical heart valves. The On-X aortic valve is currently the only mechanical heart valve approved for a lower (1.5-2.0) target international normalized ratio (INR) compared to the standard INR target 2.0-3.0. There is limited evidence demonstrating clinical benefits of the On-X valve over other mechanical aortic valves. We therefore investigated the risk of bleeding and thromboembolic events in patients with the On-X aortic valve.

**METHODS:**

This nationwide, population-based cohort study, using the target trial emulation framework, included all adults who underwent primary mechanical aortic valve replacement in Sweden 2014-2022 from the SWEDEHEART register. The rates of major bleeding and thromboembolic events were obtained from national registers. Confounding was addressed by weighting.

**RESULTS:**

Of the 3047 patients, 656 patients (22%) received an On-X aortic valve and 2391 patients (78%) received other mechanical aortic valves. The mean age was 54 years and 23% were women. After 8 years, the weighted major bleeding cumulative incidence was 7.2% (95% CI: 4.8%-10.7%) in the On-X valve group vs 7.0% (95% CI: 5.5%-8.8%) in the other mechanical valves group, and the weighted cumulative incidence of thromboembolic events was 7.3% (95% CI: 5.3%-10.2%) in the On-X valve group vs 6.4% (95% CI: 5.0%-7.9%) in the other mechanical valves group.

**CONCLUSIONS:**

We found no clinically relevant difference in major bleeding or thromboembolic events in patients with the On-X aortic valve compared to patients with other mechanical aortic valves.

## GRAPHICAL ABSTRACT

**Figure:**
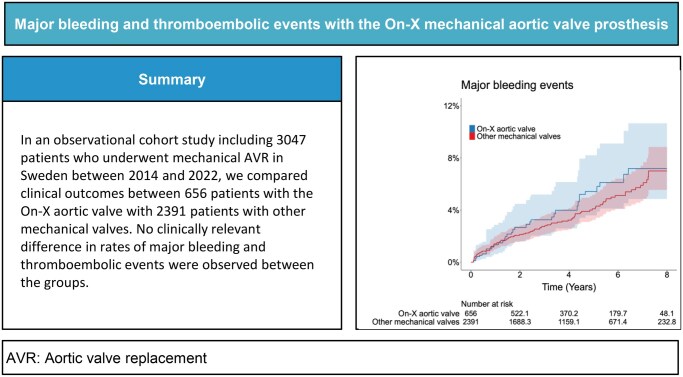

## INTRODUCTION

Mechanical heart valves have long durability but require lifelong anticoagulation therapy. Warfarin provides excellent protection against thromboembolic events in patients with mechanical valves but has a narrow therapeutic window and numerous food and drug interactions, therefore requiring frequent blood monitoring.^1^ During the last decade the use of mechanical valves has decreased.^2–4^ To address the limitations associated with warfarin while maintaining a low risk of thromboembolism, 3 different approaches have been explored: (1) the use of direct-acting oral anticoagulants (DOACs), (2) dual-antiplatelet therapy (DAPT), and (3) warfarin with a lower target international normalized ratio (INR).^2^^,^^5–8^ The first 2 strategies were unsuccessful in multiple studies, which showed a significant increase in thromboembolic and bleeding risks.^2^^,^^7^^,^^8^ To date, only the strategy of using warfarin with a lower target INR (1.5-2.0) has successfully reduced bleeding without increasing the thromboembolic risk in patients with an On-X aortic valve (On-X Life Technologies, Austin, Texas).^5^^,^^9^^,^^10^ In 2014, the Prospective Randomized On-X Anticoagulation Clinicial Trial (PROACT) study found that patients who received the On-X aortic valve with a lower target INR range of 1.5-2.0 experienced a 62% reduction in bleeding rates and similar thromboembolic event rates compared to those monitored with a standard INR range of 2.0-3.0.^5^ Based on these findings, the On-X aortic valve was approved for use with a lower target INR of 1.5-2.0, combined with a daily aspirin dose of 75-100 mg, starting 3 months after surgical aortic valve replacement (SAVR).^11^^,^^12^ After approval, data are scarce on clinical outcomes associated with the On-X aortic valve.^9^^,^^10^ We therefore conducted this nationwide, observational cohort study, applying the target trial emulation framework, to investigate the risk of bleeding and thromboembolic events in patients after On-X aortic valve replacement.

## METHODS

This study was approved by the Swedish Ethical Review Authority and the requirement for informed consent was waived. The STROBE (Strengthening and Reporting of Observational Studies in Epidemiology) and RECORD (Reporting of studies Conducted using Observational Routinely collected health Data) guidelines were followed when performing this study.^13^^,^^14^

### Study design and specification of the target trial

A hypothetical target trial was designed to address whether patients who underwent aortic valve replacement with the On-X aortic valve would have less bleeding and similar incidence of thromboembolic events compared to patients who received other mechanical aortic valves and whether this effect was causal.^15^ The major components of the target trial protocol are reported in **Table S1**. A thorough description of this approach is described in **Supplemental Methods**.

### Study population

We included all patients aged ≥18 years who underwent primary mechanical SAVR with or without concomitant coronary artery bypass grafting and ascending aortic surgery in Sweden between January 1, 2014 and December 31, 2022. Patients were excluded if they had undergone prior cardiac surgery or concomitant mechanical mitral valve replacement (**Figure S1**). **Table S2** shows the valve models and their frequency of use. The standard recommendation after aortic valve replacement with a mechanical valve prosthesis in Sweden is warfarin treatment with an INR target of 2.0-3.0. Patients who receive an On-X aortic valve prosthesis may switch to a lower target INR of 1.5-2.0, combined with a daily aspirin dose of 75 mg, 3 months post-operatively. In Europe, the On-X aortic valve was approved for use with lower target INR in January 2014. In this study, we did not have information on the quality of warfarin monitoring because INR values were not available.

### Outcomes

The primary outcomes of interest were major bleeding events and thromboembolic events. The secondary outcomes were all-cause mortality and aortic valve reintervention. All outcomes were identified from the Swedish National Patient Register^16^ according to the International Classification of Disease, 9-10th revision codes (**Table S3**).

### Data sources

Nationwide, population-based Swedish registries were used to emulate the described hypothetical target trial. Patients were identified from the Swedish Cardiac Surgery Register, which is a part of the SWEDEHEART (Swedish Web-System for Enhancement and Development of Evidence-Based Care in Heart Disease Evaluated According to Recommended Therapies) register.^17^^,^^18^ Baseline characteristics were obtained from the Swedish Cardiac Surgery Register and the Swedish National Patient Register. Survival status was obtained from the Swedish Total Population Register.^19^ Socioeconomic background characteristics were obtained from the longitudinal integrated database for health insurance and labour market studies.^20^ The Swedish personal identity number allowed cross-linking of data from these registries for each individual.^21^

### Statistical methods

Categorical baseline characteristics were presented as frequencies (percentages) and continuous variables were presented as means (standard deviations). To emulate randomization, the primary analysis method used was stabilized optimization-based weights to balance the groups with respect to all variables in **Table 1**. As secondary and sensitivity analysis, to confirm the results obtained from optimization-based weights, we used overlap weights. Overlap weights, derived from propensity scores estimated using a logistic regression model, were applied to focus the analysis on the overlapping population between the exposure and control groups (representing clinical equipoise) while ensuring that no study participants were excluded from the available sample.^22^ The time to event was defined as the day of surgery until the date of the event or the end of follow-up (December 31, 2022), whichever occurred first. The median follow-up time was derived based on the reverse Kaplan-Meier method. The Aalen-Johansen estimator was used to estimate the crude cumulative incidence of major bleeding events, thromboembolic events, and aortic valve reintervention while accounting for the competing risk of death. The crude cumulative incidence of survival was calculated using the Kaplan-Meier method. Data management and statistical analyses were performed using the R programming language, Version 4.4.2 (R Foundation for Statistical Computing) using the “WeightIt” package.

**Table 1.** Baseline Characteristics of Patients Who Received an On-X Aortic Mechanical Valve or Other Mechanical Valves in Sweden Between 2014 and 2022

Variable	Overall	On-X	Other mechanical	*P*-value
No.	3047	656 (22%)	2391 (78%)	
Age, years (mean [SD])	53.5 (10.6)	52.9 (10.8)	53.6 (10.6)	.124
Age, years (median [IQR])	55.0 [48.0, 61.0]	55.0 [47.8, 60.0]	56.0 [49.0, 61.0]	.284
Women	712 (23.4)	116 (17.7)	596 (24.9)	<.001
Married	1563 (51.3)	351 (53.5)	1212 (50.7)	.217
Education level				.026
<10 years	489 (16.1)	86 (13.1)	403 (17.0)	
10-12 years	1585 (52.3)	368 (56.2)	1217 (51.3)	
>12 years	955 (31.5)	201 (30.7)	754 (31.8)	
Non-Nordic birth region	334 (11.0)	37 (5.6)	297 (12.4)	<.001
Household income				.174
*Q*1 (lowest)	762 (25.0)	160 (24.4)	602 (25.2)	
*Q*2	762 (25.0)	146 (22.3)	616 (25.8)	
*Q*3	762 (25.0)	170 (25.9)	592 (24.8)	
*Q*4 (highest)	761 (25.0)	180 (27.4)	581 (24.3)	
Body mass index, kg/m^2^				.636
<18.5	25 (0.8)	4 (0.6)	21 (0.9)	
18.5-24.9	816 (27.3)	189 (28.9)	627 (26.8)	
25-29.9	1197 (40.0)	261 (39.8)	936 (40.0)	
>30	956 (31.9)	201 (30.7)	755 (32.3)	
Diabetes mellitus	353 (11.6)	72 (11.0)	281 (11.8)	.630
Prior atrial fibrillation	346 (11.4)	66 (10.1)	280 (11.7)	.267
Hypertension	1309 (43.0)	288 (43.9)	1021 (42.7)	.613
Hyperlipidemia	496 (16.3)	104 (15.9)	392 (16.4)	.785
Prior stroke	196 (6.4)	44 (6.7)	152 (6.4)	.815
Peripheral vascular disease	25 (0.8)	5 (0.8)	20 (0.8)	1.000
COPD	73 (2.4)	10 (1.5)	63 (2.6)	.133
Prior myocardial infarction	149 (4.9)	36 (5.5)	113 (4.7)	.484
Prior PCI	159 (5.2)	43 (6.6)	116 (4.9)	.101
Prior major bleeding event	338 (11.1)	65 (9.9)	273 (11.4)	.308
Alcohol dependence	117 (3.8)	19 (2.9)	98 (4.1)	.192
Hepatic disease	39 (1.3)	8 (1.2)	31 (1.3)	1.000
History of cancer	226 (7.4)	43 (6.6)	183 (7.7)	.386
eGFR, mL/min/1.73 m^2^				.432
>60	2817 (92.8)	617 (94.2)	2200 (92.4)	
45-60	134 (4.4)	24 (3.7)	110 (4.6)	
30-44	40 (1.3)	5 (0.8)	35 (1.5)	
15-29	15 (0.5)	2 (0.3)	13 (0.5)	
<15	31 (1.0)	7 (1.1)	24 (1.0)	
Preoperative dialysis	23 (0.8)	5 (0.8)	18 (0.8)	1.000
Pacemaker/ICD	60 (2.0)	18 (2.7)	42 (1.8)	.146
Preoperative heart failure	490 (16.1)	111 (16.9)	379 (15.9)	.548
LVEF				.757
>50%	2283 (75.0)	490 (74.8)	1793 (75.1)	
30%-50%	612 (20.1)	136 (20.8)	476 (19.9)	
<30%	149 (4.9)	29 (4.4)	120 (5.0)	
Prior endocarditis	76 (2.5)	18 (2.7)	58 (2.4)	.748
Emergent operation	90 (3.0)	12 (1.8)	78 (3.3)	.073
Isolated AVR	1658 (54.4)	410 (62.5)	1248 (52.2)	<.001
Concomitant CABG	353 (11.6)	80 (12.2)	273 (11.4)	.630
Ascending aortic surgery	1127 (37.0)	185 (28.2)	942 (39.4)	<.001
DHCA	232 (7.6)	23 (3.5)	209 (8.7)	<.001

Numbers are *N* and (%) unless otherwise stated.

Abbreviations: AVR, aortic valve replacement; CABG, coronary artery bypass graft surgery; COPD, chronic obstructive pulmonary disease; DHCA, deep hypothermic circulatory arrest; eGFR, estimated glomerular filtration rate; ICD, implantable cardioverter-defibrillator; IQR, interquartile range; LVEF, left ventricular ejection fraction; PCI, percutaneous coronary intervention; *Q*1-*Q*4, quartiles 1-4; SD, standard deviation

### Missing data

There was a low rate of missing baseline data. The variables with missing data were body mass index (1.7%), education level (0.6%), estimated glomerular filtration rate (0.3%), left ventricular ejection fraction (0.1%), and preoperative dialysis (0.1%). Missing data were handled by the classification and regression tree estimated and imputation approach, which is a machine learning technique providing predictions based on all non-missing data (see also **Supplementary Methods**).

## RESULTS

In total, we included 3047 patients who underwent primary SAVR with a mechanical prosthesis in Sweden between 2014 and 2022. Of these, 656 patients (22%) received an On-X aortic valve and 2391 patients (78%) received other mechanical aortic valves. **Table 1** shows the baseline characteristics of the 2 groups. The mean age was 54 years and 23% were women. Patients who received the On-X aortic valve were less often women, and had less concomitant ascending aortic surgery. Most baseline characteristics were similar between the groups. **Tables S4** and **S5** and **Figure S2** show the frequency of On-X aortic prosthesis implantation per year and hospital. The baseline characteristics before and after optimization-based weighting and overlap weighting are presented in **Tables S6** and **S7** and Figures S3 and S4. After weighting with either method, the measured covariates were well balanced between patients with the On-X aortic valve and those with other mechanical valves.

### Clinical outcomes

The crude cumulative incidence of all outcomes is shown in **Table S9** and **Figures S5-S8**. The adjusted cumulative incidence of major bleeding events, thromboembolic events, all-cause mortality, and aortic valve reintervention is presented in **Table S8**. The median follow-up time by group is presented in **Table S10**.

### Major bleeding events

**Figure 1.**
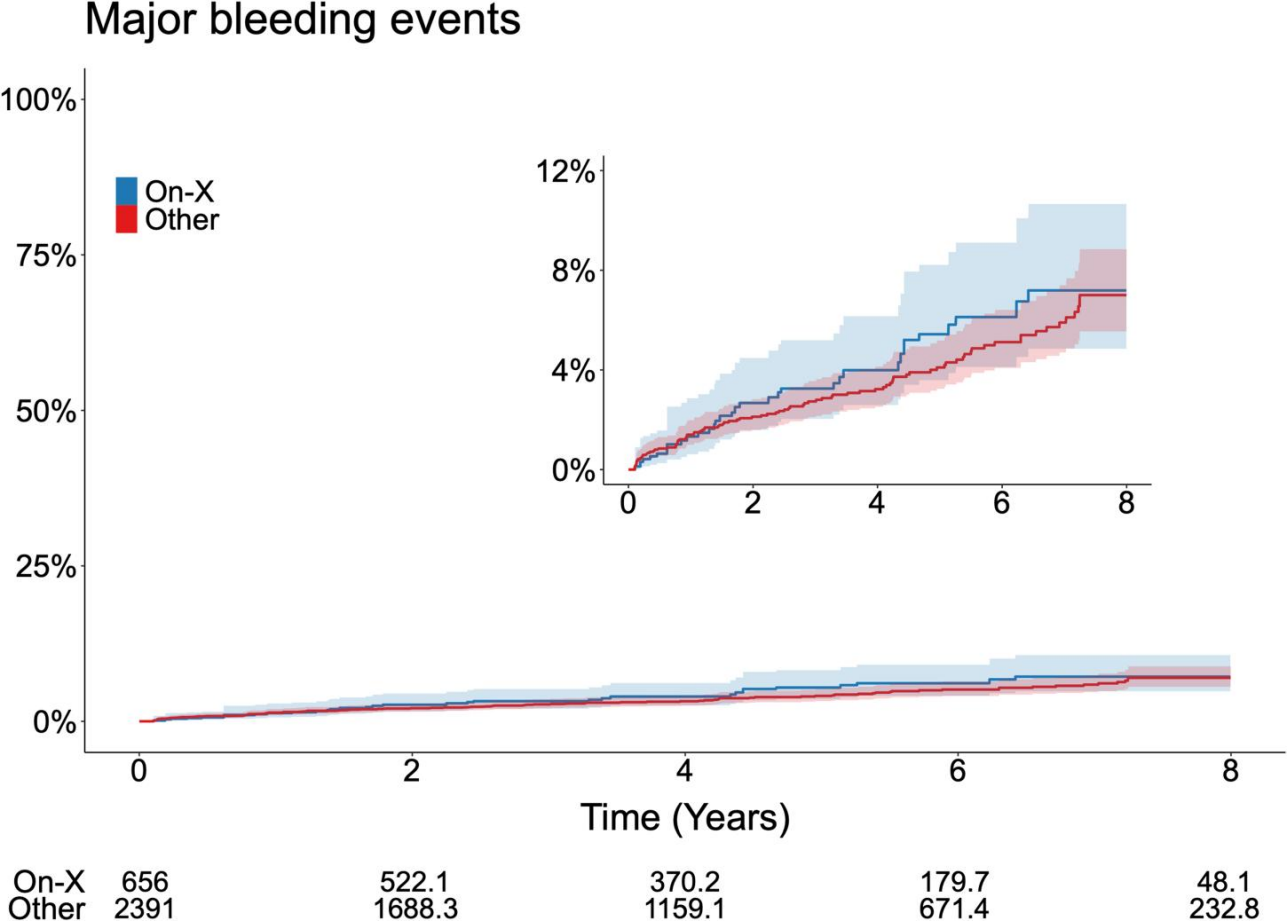
Major Bleeding Events. Adjusted Cumulative Incidence of Major Bleeding in Patients with On-X Aortic Valve or Other Mechanical Valves after SAVR in Sweden between 2014 and 2022

In total, a first-time bleeding event occurred in 122 patients (4.0%). **Table S11** shows the frequently of first- and multiple-time bleeding in the study population. At 8 years of follow-up, the weighted cumulative incidence of major bleeding events was 7.2% (95% CI: 4.8%-10.7%) in the On-X valve group compared to 7.0% (95% CI: 5.5%-8.8%) in the other mechanical valves group (**Table S8** and **Figure 1**).

### Thromboembolic events

**Figure 2.**
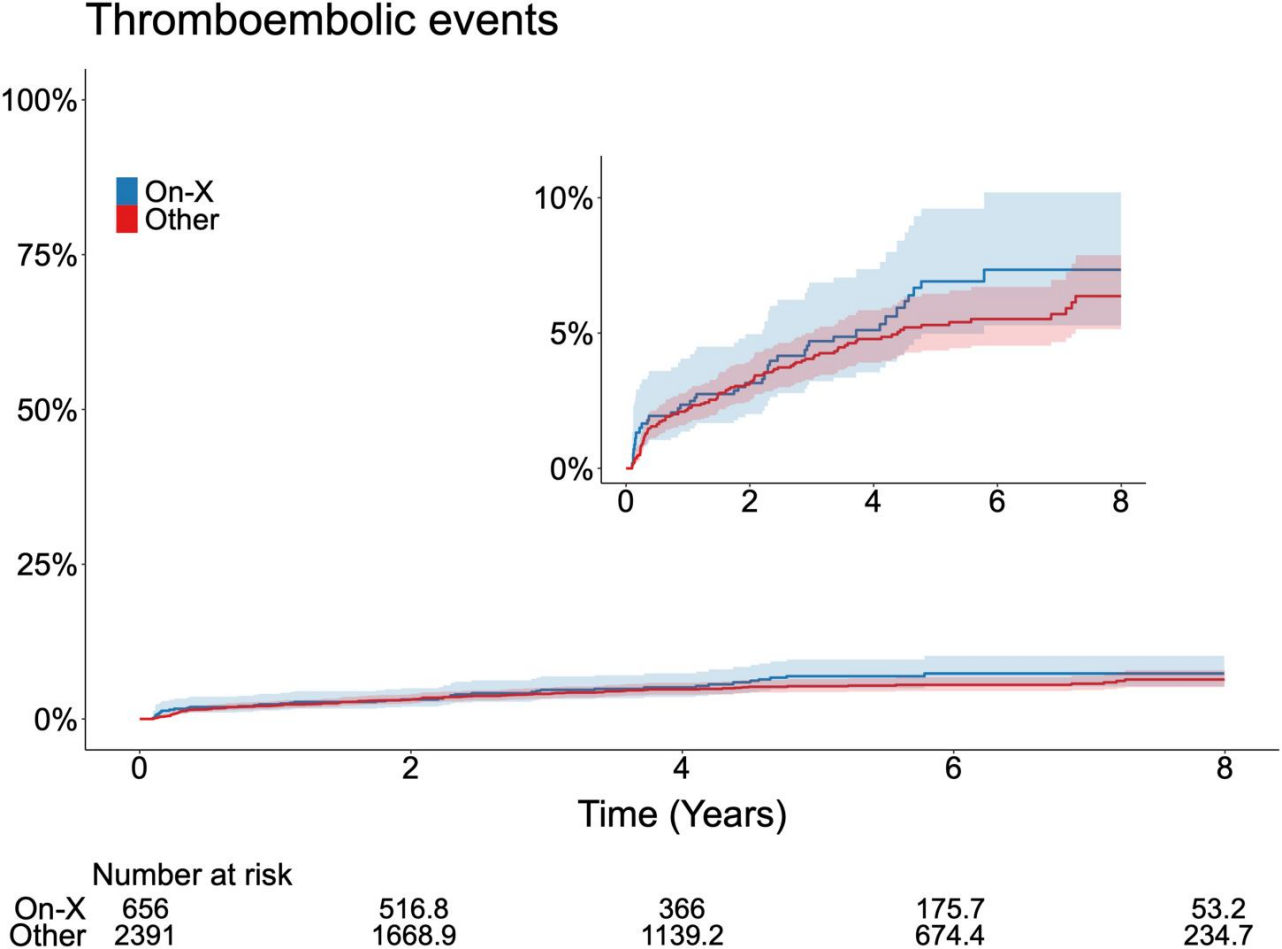
Thromboembolic Events. Adjusted Cumulative Incidence of Thromboembolic Events in Patients with On-X Aortic Valve or Other Mechanical Valves after SAVR in Sweden Between 2014 and 2022

In total, a first-time thromboembolic event occurred in 150 patients (6.3%). At 8 years of follow-up, the weighted cumulative incidence of thromboembolic events was 7.3% (95% CI: 5.3%-10.2%) in the On-X valve group and 6.4% (95% CI: 5.0%-7.9%) in the other mechanical valves group (**Table S8** and **Figure 2**).

### Survival

In total, 155 patients (5.0%) died. At 8 years of follow-up, the weighted cumulative incidence of all-cause mortality was 7.0% (95% CI: 4.6%-10.6%) in the On-X valve group and 9.6% (95% CI: 8.0%-11.8%) in the other mechanical valves group (**Table S8** and **Figure S9**).

### Aortic valve reintervention

In total, 55 patients (1.8%) underwent an aortic valve reintervention. After weighting, there was no significant difference between the groups (**Table S8** and **Figure S10**).

### Sensitivity and subgroup analyses

Additional analyses using overlap weighting, as well as separate subgroup analyses of patients without prior atrial fibrillation, patients without prior bleeding events, and patients who underwent isolated AVR, were consistent with the results of the main analyses (**Figures S11-S23**).

## DISCUSSION

In this nationwide cohort study, we observed no significant difference in major bleeding or thromboembolic events in patients with the On-X aortic valve vs other mechanical aortic valves up to 9 years of follow-up. The rates of all-cause mortality and aortic valve reintervention were also similar between the groups.

For mechanical valves, the primary objective is to develop a therapeutic approach that provides a delicate balance between preventing thromboembolism and minimizing bleeding. Although warfarin has well-documented limitations, it remains the only approved treatment for patients with mechanical valves.^11^^,^^12^ Wang et al conducted a non-inferiority trial enrolling 863 patients, comparing thromboembolic risks between patients who were randomized to apixaban or warfarin after SAVR using the On-X valve.^7^ This trial was terminated early due to the increased thromboembolic risks associated with apixaban compared to warfarin (rate difference: 2.9; 95% CI: 0.8-5.0). Eikelboom and colleagues conducted a randomized trial validating a novel dabigatran regimen for preventing thromboembolic complications in patients with mechanical valves.^8^ This trial was also terminated early after enrolling 252 patients due to higher thromboembolic (HR 3.37; 95% CI: 0.76-14.95) and bleeding rates (HR 2.45; 95% CI: 1.23-4.86) in the dabigatran group compared to the warfarin group. The early discontinuation observed in trials evaluating DOACs may be attributed to suboptimal plasma drug levels and pharmacodynamic mechanisms distinct from those of warfarin.^8^ Although increasing the DOAC dose could theoretically reduce thromboembolic risk, it would also increase the bleeding risk. The randomized trial by Puskas et al evaluating the safety of DAPT in patients with an On-X aortic valve was discontinued prematurely due to significantly increased cerebral thromboembolic events in the DAPT group compared to the warfarin group (3.12%/patient-year vs 0.29/patient-years, *P* = .02).^2^ The only strategy proven to reduce bleeding risk without increasing thromboembolic events following mechanical SAVR is the use of a lower target INR range, as shown by Torella et al and Puskas et al.^5^^,^^6^^,^^11^^,^^12^

Few studies have investigated the clinical outcomes of a lower target INR range in patients with the On-X aortic valve after the FDA approval.^9^^,^^10^ Oo et al analysed the long-term risks of thromboembolism and major bleeding in 510 patients from 2015 to 2022 who received the On-X aortic valve with lower target INR range (1.5-2.0) compared to the control group in the PROACT study (INR 2.0-3.0).^9^ They found 73% lower rates of bleeding in patients targeted with a lower INR range compared to the PROACT control group (linearized occurrence rate: 1.9% vs 7.1%) and similar thromboembolic risks (linearized occurrence rate: 1.7% vs 1.4%).^9^ While that study was well-conducted, its reliance on historical comparisons may introduce bias, as different time frames result in non-comparable groups, thereby limiting its clinical relevance. To the best of our knowledge, no study has compared the long-term clinical outcomes of the On-X aortic valve to other mechanical aortic valves after the FDA approval.

The novel design of the On-X aortic valve is unique, and currently it is the only mechanical aortic valve approved for use with a lower target INR range.^11^^,^^12^ The On-X valve is constructed with a new generation pure pyrolytic carbon, free of silicon, which is known to serve as a nidus for platelet aggregation and subsequent thrombus formation in conventional mechanical valves.^23^ The key features of the On-X valve promote laminar flow and reduce thrombogenicity.^24^

A possible explanation for the similar rates of bleeding and thromboembolic events in the groups could be that the regimen of anticoagulation was similar. Even though the On-X aortic valve is approved for use with a lower target INR range, it is possible that many patients with the On-X aortic valves were kept on the standard recommendation after SAVR with a mechanical valve prosthesis with an INR target of 2.0-3.0. If that was the case, the potential advantage of the On-X aortic valve—a lower INR target range and presumably reduced bleeding risk—would be omitted. We were not able to analyse the INR values or time within therapeutic range, because we lacked these data. Nevertheless, our study investigated the consequences of a strategy of SAVR with the On-X valve on bleeding rates, and found that the bleeding rates were not different from those observed in patients who received other contemporary mechanical valves. Further studies should include INR values to improve our understanding of bleeding risk and thromboembolism in patients with mechanical aortic heart valves. Moreover, it is unclear to what extent healthcare providers offering anticoagulation treatment are aware of the option to lower the target INR range for patients with the On-X valve. Implementing informational interventions to raise awareness may therefore be beneficial. Evidence behind guideline recommendations for anticoagulation following mechanical SAVR is weak, and a recent global survey showed that anticoagulation care and INR monitoring varied between clinicians.^25^

In the absence of a randomized clinical trial directly comparing On-X aortic valves with other mechanical aortic valves following the FDA approval, this observational emulation of a target trial offers evidence to guide clinical decision-making. Our study found no clinically relevant difference in major bleeding and thromboembolic events between the On-X aortic valve and other mechanical aortic valves.

### Strengths and limitations

The strengths of our study include the large contemporary study population and the complete and accurate follow-up owing to the high quality of the national Swedish registries. We were able to link information from several high quality and complete health data registers in Sweden. In Sweden, the quality of anticoagulation monitoring has repeatedly been proven to be very high.^26^^,^^27^ While this study did not involve the randomization of patients to specific valve types, the allocation of treatment (ie, valve choice) was based on surgeon or centre preference. It is therefore reasonable to believe that the valve choice was independent of any specific patient characteristics, making it comparable to a natural experiment or pseudo-randomized study. The crude results should be fairly free from confounding, and this assumption was corroborated by the fact that the unadjusted and adjusted analyses gave similar results. Although this was an observational study, it is likely that the risk of residual confounding was minimal.

This study had several important limitations. The national registries used did not include data on the quality of warfarin monitoring. We did not have information on INR values or time within therapeutic range. We cannot ascertain whether patients with the On-X aortic valve were in fact monitored with a lower target INR range or a standard INR range. We lack information on whether INR was home or clinic-monitored. Another limitation was that the definition of major bleeding in our study did not capture minor bleeding events, which could have affected patient well-being and contributed to a lower quality of life.

## CONCLUSIONS

We found no clinically relevant difference in major bleeding or thromboembolic events in patients who received the On-X aortic valve compared to patients with other mechanical aortic valves. Future studies including INR values are needed to confirm whether lower INR ranges can improve clinical outcomes following aortic valve replacement.

## Supplementary Material

ivaf182_Supplementary_Data

## Data Availability

Data cannot be shared for ethical/privacy reasons. The data underlying this article cannot be shared publicly due to privacy of individuals that were included.
