# Supplementary material for: Major Bleeding and Thromboembolic Events with the On-X Mechanical Aortic Valve Prosthesis: A SWEDEHEART Study
Source: Interdiscip Cardiovasc Thorac Surg. 2025 Jul 29;40(8):ivaf182. doi: 10.1093/icvts/ivaf182 (PMC12342359; doi:10.1093/icvts/ivaf182)

**Major bleeding and thromboembolic events in patients with the On-X mechanical aortic valve prostheses**

***Running title: Clinical outcomes of the On-X aortic valve***

Ruixin Lu^1^, MD, Michael Dismorr^1^, MD, PhD, Magnus Dalén, MD, PhD^1,2^, Natalie Glaser^1,3^, MD, PhD, Ulrik Sartipy, MD, PhD^1,2^

ORCIDs

Ruixin Lu: <https://orcid.org/0009-0007-8604-1565>

Michael Dismorr: https://orcid.org/0000-0002-5652-8774

Magnus Dalén: https://orcid.org/0000-0001-5352-5132

Natalie Glaser: https://orcid.org/0000-0002-4386-1041

Ulrik Sartipy: https://orcid.org/0000-0003-2707-0263

^1^Department of Molecular Medicine and Surgery, Karolinska Institutet, Stockholm, Sweden

^2^Department of Cardiothoracic Surgery, Karolinska University Hospital, Stockholm, Sweden

^3^Department of Cardiology, Stockholm South General Hospital, Stockholm, Sweden

**Corresponding author:**

Ruixin Lu

Department of Molecular Medicine and Surgery

Karolinska Institutet

SE-171 77 Stockholm, Sweden

E-mail: *Ruixin.Lu@ki.se*

*Table of Contents*

[Supplemental Methods: Statistical methods 3](#_Toc199073189)

[*Target trial emulation 3*](#_Toc199073190)

[*Major principles of target trial emulation 3*](#_Toc199073191)

[*Importance of target trial emulation 3*](#_Toc199073192)

[*Classification and regression tree imputation (CART) 4*](#_Toc199073193)

[Supplemental References 5](#_Toc199073194)

[Supplemental Table S1. Target Trial Protocol, comparison between target trial and emulation 6](#_Toc199073195)

[Supplemental Table S2. Frequencies of different On-X aortic and aortic mechanical valves 9](#_Toc199073196)

[Supplemental Table S3. ICD 9 and 10 codes 10](#_Toc199073197)

[Supplemental Table S4. Number of patients by year of surgery 11](#_Toc199073198)

[Supplemental Table S5. Number of patients according to hospital 12](#_Toc199073199)

[Supplemental Table S6. Baseline characteristics after optimization-based weighting. 13](#_Toc199073200)

[Supplemental Table S7. Baseline characteristics after overlap weighting 16](#_Toc199073201)

[Supplemental Table S8. Cumulative incidence of clinical outcomes in the weighted population 19](#_Toc199073202)

[Supplemental Table S9. Crude cumulative incidence for major bleeding events, thromboembolic events, all-cause mortality, and aortic valve reintervention 20](#_Toc199073203)

[Supplemental Table S10. Median follow-up time in patients who underwent mechanical aortic valve replacement in Sweden between 2014 and 2022, by group. 21](#_Toc199073204)

[Supplemental Table S11. Frequencies of bleeding events 22](#_Toc199073205)

[Supplemental Table S12. Number of (first time) events 23](#_Toc199073206)

[Supplemental Figure S1: Flowchart of study population 24](#_Toc199073207)

[Supplemental Figure S2: Proportion of On-X aortic valves operated per year in Sweden 25](#_Toc199073208)

[Supplemental Figure S3: Absolute mean differences before (red circles) and after (blue circles) optimization-based weighting 26](#_Toc199073209)

[Supplemental Figure S4: Absolute mean differences before (red circles) and after (blue circles) overlap weighting 27](#_Toc199073210)

[Supplemental Figure S5: Aalen-Johansen estimated crude cumulative incidence of major bleeding events 28](#_Toc199073211)

[Supplemental Figure S6: Aalen-Johansen estimated crude cumulative incidence of thromboembolic events 29](#_Toc199073212)

[Supplemental Figure S7: Kaplan-Meier estimated crude survival 30](#_Toc199073213)

[Supplemental Figure S8: Aalen-Johansen estimated crude cumulative incidence of aortic valve reintervention 31](#_Toc199073214)

[Supplemental Figure S9: Cumulative incidence of survival after optimization-based weighting 32](#_Toc199073215)

[Supplemental Figure S10: Cumulative incidence of aortic valve reintervention after optimization-based weighting 33](#_Toc199073216)

[Supplemental Figure S11: Cumulative incidence of major bleeding events after overlap weighting 34](#_Toc199073217)

[Supplemental Figure S12: Cumulative incidence of thromboembolic events after overlap weighting 35](#_Toc199073218)

[Supplemental Figure S13: Cumulative incidence of survival after overlap weighting 36](#_Toc199073219)

[Supplemental Figure S14: Cumulative incidence of aortic valve reintervention after overlap weighting 37](#_Toc199073220)

[Supplemental Figure S15: Absolute mean differences before (red circles) and after (blue circles) optimization-based weighting in patients without preoperative atrial fibrillation 38](#_Toc199073221)

[Supplemental Figure S16: Cumulative incidence of survival after optimization-based weighting in patients without preoperative atrial fibrillation 39](#_Toc199073222)

[Supplemental Figure S17: Cumulative incidence of major bleeding events and thromboembolic events after optimization-based weighting in patients without preoperative atrial fibrillation 40](#_Toc199073223)

[Supplemental Figure S18: Absolute mean differences before (red circles) and after (blue circles) optimization-based weighting in patients without preoperative major bleeding events 41](#_Toc199073224)

[Supplemental Figure S19: Cumulative incidence of survival after optimization-based weighting in patients without preoperative major bleeding events 42](#_Toc199073225)

[Supplemental Figure S20: Cumulative incidence of major bleeding events and thromboembolic events after optimization-based weighting in patients without preoperative major bleeding events 43](#_Toc199073226)

[Supplemental Figure S21: Absolute mean differences before (red circles) and after (blue circles) optimization-based weighting in patients with underwent isolated AVR 52](#_Toc199073227)

[Supplemental Figure S22: Cumulative incidence of survival after optimization-based weighting in patients who underwent isolated AVR 52](#_Toc199073228)

[Supplemental Figure S23: Cumulative incidence of major bleeding events and thromboembolic events after optimization-based weighting in patients without preoperative major bleeding events 52](#_Toc199073229)

[Supplemental Figure S24: Age distribution by valve group. 52](#_Toc199073230)

# **Supplemental Methods: Statistical methods**

## *Target trial emulation*

This study used the target trial emulation framework. Observational studies can provide evidence on the effect of treatment when randomized studies are not feasible, either due to ethical reasons, high economical costs or taking too long time. Observational studies, however, are not randomized and thus cannot provide causal relationships between the exposure and the outcome due to confounding bias. Incorrect study design choices in observational studies can often lead to self-inflicted biases, causing the results to be less reliable.^1^ These study design flaws may be eliminated by performing a hypothetical randomized trial, i.e. the target trial, to answer the question of interest. First, a target trial protocol is designed by applying the principles of randomization. Then, this trial is emulated by using observational data together with appropriate methodology, such as inverse probability of treatment weighting.^2^

## *Major principles of target trial emulation*

The first major principle of target trial emulation is that the treatment of interest should not occur before the follow-up time, therefore prevalent users should be excluded at or before baseline. The second major principle is that immortal time bias should be avoided, the exposure should be defined at the start of follow-up (time zero), not after some time into the follow-up.^3^

## *Importance of target trial emulation*

Observational studies are prone to confounding despite adjustment for potential confounders and analytical method used. Nonetheless, with a successfully emulated target trial the observational data will provide the same effect estimates as data from a randomized trial.^3^

## *Classification and regression tree imputation (CART)*

The Classification and regression tree (CART) is a popular machine learning technique used for imputation of missing data developed by Breiman et al in 1984. The imputation approach predicts missing data based on all available (non-missing) data. For discrete missing variables, the CART algorithm identifies the outcome group based on Gini impurity. For continuous missing variables, the CART algorithm predicts missing using a regression model and performs splits in the tree based on minimizing the residual sum of squares.

Binary, categorical, and continuous variables can be used by the CART algorithm as predictors and as results. This algorithm is robust against outliers. It is implemented in the R packages *rpart, simputation,* and *mice.* For further reading, please read “Buuren (ISBN-13: 978-1138588318)”.^4^

# **Supplemental References**

1. Huitfeldt A, Hernan MA, Kalager M, Robins JM. Comparative Effectiveness Research Using Observational Data: Active Comparators to Emulate Target Trials with Inactive Comparators. *EGEMS Wash DC*. 2016;4:1234.

2. Dickerman BA, García-Albéniz X, Logan RW, Denaxas S, Hernán MA. Avoidable flaws in observational analyses: an application to statins and cancer. *Nat Med*. 2019;25:1601–1606.

3. Matthews AA, Danaei G, Islam N, Kurth T. Target trial emulation: applying principles of randomised trials to observational studies. *BMJ*. 2022;:e071108.

4. Buuren S. Flexible Imputation of Missing Data, 2nd Edition. CRC Press; 2018.

5. Xue X, Agalliu I, Kim MY, Wang T, Lin J, Ghavamian R, et al. New methods for estimating follow-up rates in cohort studies. BMC Med Res Methodol 2017;17:155. https://doi.org/10.1186/s12874-017-0436-z.

| **Supplemental Table S1. Target Trial Protocol, comparison between target trial and emulation.** | | | |
| --- | --- | --- | --- |
| **Protocol component** | **Target trial specification** | **Target trial emulation** | **Data sources for emulation** |
| **Aim** | To investigate the risks of major bleeding, thromboembolic events, all-cause mortality and aortic valve reintervention risks of the On-X aortic valve compared with other mechanical aortic valves. | Same. | – |
| **Eligibility criteria** | - Implantation of mechanical AVR. - Adults, age ≥ 18. - No prior cardiac surgery. | Same. | - Swedish Cardiac Surgery Register. - National Patient Register. |
| **Enrollment** | 2014-01-01 – 2022-12-31 | Same. | - Swedish Cardiac Surgery Register. |
| **Treatment strategies** | 1. SAVR with On-X aortic valve. 2. SAVR with all other mechanical aortic valves except the On-X aortic valve. | Same. | - Swedish Cardiac Surgery Register. |
| **Treatment assignment** | - Randomly assigned in a 1:1 ratio to receive either the On-X aortic mechanical valve or other mechanical aortic valves. - Both surgeons and patients will be aware of the assigned valve model. | - Patients were classified according to the type of valve they received during surgery (SAVR), exposure was defined at the time of surgery. - Assignment according to surgeon or center preference. | - Swedish Cardiac Surgery Register. |
| **Outcomes** | - Major bleeding events. - Thromboembolic events/stroke/TIA. - All-cause mortality. - Aortic valve reintervention. - Obtained from ICD-9/10 codes. | Same. | - National Patient Register. - Total Population Register. - SWENTRY register. - Swedish Cardiac Surgery Register. |
| **Follow-up** | - **Intention-to-treat:** starts on day of SAVR and ends at day of death, major bleeding event, aortic valve reintervention or 31 December 2022, whichever occurs first. | Same.  Patients who, for example, “drop out” from the On-X aortic valve group and receive a different type of mechanical aortic valve (other than the On-X aortic prosthesis) are classified under the outcome “aortic valve reintervention”. No per-protocol analysis has been conducted. | - Swedish Cardiac Surgery Register. - National Patient Register. - SWENTRY register. |
| **Causal estimand** | Intention-to-treat. | Observational analogue of the intention-to-treat effect. | – |
| **Statistical analyses** | - Intention-to-treat analysis. - No statistical analysis will be used for adjusting for potential confounding covariates at baseline, since the trial is randomized. - Pearson’s chi-squared test. - Subgroup analyses on patients without prior atrial fibrillation, patients without prior bleeding events, and patients who underwent isolated SAVR. | - Same intention-to-treat analysis and additional adjustment for baseline covariates. - Stabilized optimization-based weights were used to balance the groups. - Overlap weights. - Missing data were handled using the Classification and Regression tree estimation and imputation approach. - Same subgroup analyses. | – |
| ICD = International Classification of Diseases. SAVR = Surgical aortic valve replacement. SWENTRY = Swedish Transcatheter Cardiac Intervention Registry. | | | |

| **Supplemental Table S2. Frequencies of different On-X aortic and aortic mechanical valves used in patients between 2014 and 2022.** | |
| --- | --- |
| **Model** | **No.** |
| **On-X aortic valve** | |
| On-X aortic valve | 656 |
| **Other mechanical valves** | |
| Carbomedics | 991 |
| Regent | 659 |
| Masters | 524 |
| Open Pivot | 170 |
| Other | 47 |
|  | |

| **Supplemental Table S3. ICD 9 and 10 codes used to identify outcomes and exposure in patients who underwent mechanical aortic valve replacement in Sweden between 2014 and 2022.** | |
| --- | --- |
| **Outcome and exposure** | **ICD 10 codes** |
| **Major bleeding events** | I60, I61, I62, I850, K226, K250, K252, K254,  K256, K260, K262, K264, K266, K270, K272, K274, K276,  K280, K282, K284, K286, K290, K625, K920, K921, K922,  D629, D500, R040, R041, R042, R048, R049, R319, I312, J942 |
| **Thromboembolic events** | I63, I64, I74  G458, G459 |
| **Aortic valve reintervention** | FMD00, FMD10, FMD12, FMD13, FMD30,  FMD33, FMD96, FCA60, FCA70 |
| ICD = International Classification of Diseases | |

| **Supplemental Table S4. Number of patients by year of surgery between 2014 and 2022.** | | | |
| --- | --- | --- | --- |
| **Year of Surgery** | **Total** | **On-X aortic valve** | **Other mechanical valves** |
|  | 3,047 | 656 | 2391 |
| **2014** | 296 | 53 (18%) | 243 (82%) |
| **2015** | 271 | 50 (18%) | 221 (82%) |
| **2016** | 348 | 90 (26%) | 258 (74%) |
| **2017** | 311 | 81 (26%) | 230 (74%) |
| **2018** | 358 | 97 (27%) | 261 (73%) |
| **2019** | 321 | 85 (26%) | 236 (74%) |
| **2020** | 352 | 75 (21%) | 277 (79%) |
| **2021** | 391 | 69 (18%) | 322 (82%) |
| **2022** | 399 | 56 (14%) | 343 (86%) |
| Numbers are N and (%) unless otherwise stated. | | | |

| **Supplemental Table S5. Number of patients according to hospital between 2014 and 2022.** | | | |
| --- | --- | --- | --- |
| **Hospital** | **Total** | **On-X aortic valve** | **Other mechanical valves** |
|  | 3,047 | 656 | 2391 |
| **A** | 330 | 115 (35%) | 215 (65%) |
| **B** | 791 | 101 (13%) | 690 (87%) |
| **C** | 345 | 80 (23%) | 265 (77%) |
| **D** | 590 | 357 (61%) | 233 (39%) |
| **E** | 221 | 3 (1%) | 218 (99%) |
| **F** | 426 | 0 (0%) | 426 (100%) |
| **G** | 249 | 0 (0%) | 249 (100%) |
| **H** | 95 | 0 (0%) | 95 (100%) |
| Numbers are N and (%) unless otherwise stated. | | | |

| **Supplemental Table S6. Baseline characteristics of patients who received On-X aortic mechanical valve or other mechanical valves in Sweden between 2014 and 2022 after optimization-based weighting.** | | | | | | |
| --- | --- | --- | --- | --- | --- | --- |
|  | **Before weighting, %** | | | **After weighting, %** | | |
| Variable | On-X aortic valve | Other mechanical valves | Absolute mean difference | On-X aortic valve | Other mechanical valves | Absolute mean difference |
| Age, years (mean [SD]) * | 52.9 (10.8) | 53.6 (10.6) | 0.07 | 53.3 (10.7) | 53.6 (10.6) | 0.03 |
| Female sex | 17.7 | 24.9 | 7.24 | 22.1 | 24.6 | 2.50 |
| Married | 53.5 | 50.7 | 2.82 | 52.5 | 50.6 | 1.95 |
| Education <10 years | 13.1 | 16.9 | 3.75 | 14.8 | 16.9 | 2.08 |
| Education 10-12 years | 56.1 | 51.5 | 4.57 | 53.8 | 51.5 | 2.21 |
| Education >12 years | 30.8 | 31.6 | 0.83 | 31.4 | 31.6 | 0.13 |
| Non-Nordic birth region | 5.6 | 12.4 | 6.78 | 9.7 | 12.2 | 2.50 |
| Household income Q1 (Lowest) | 24.4 | 25.2 | 0.79 | 26.3 | 25.2 | 1.07 |
| Household income Q2 | 22.3 | 25.8 | 3.51 | 23.8 | 25.8 | 2.03 |
| Household income Q3 | 25.9 | 24.8 | 1.16 | 24.4 | 24.8 | 0.34 |
| Household income Q4 (Highest) | 27.4 | 24.3 | 3.14 | 25.5 | 24.3 | 1.29 |
| BMI <18.5 | 0.6 | 0.9 | 0.27 | 0.6 | 0.9 | 0.29 |
| BMI 18.5-24.9 | 28.8 | 26.7 | 2.09 | 28.4 | 26.7 | 1.76 |
| BMI 25-29.9 | 39.9 | 40.1 | 0.13 | 39.6 | 40.1 | 0.51 |
| BMI >30 | 30.6 | 32.3 | 1.69 | 31.4 | 32.4 | 0.96 |
| Diabetes mellitus | 11 | 11.8 | 0.78 | 10.9 | 11.8 | 0.93 |
| Prior atrial fibrillation | 10.1 | 11.7 | 1.65 | 10.1 | 11.7 | 1.64 |
| Prior hypertension | 43.9 | 42.7 | 1.20 | 44.2 | 42.7 | 1.51 |
| Hyperlipidemia | 15.9 | 16.4 | 0.54 | 16.2 | 16.5 | 0.28 |
| Prior stroke | 6.7 | 6.4 | 0.35 | 6.7 | 6.4 | 0.33 |
| Peripheral vascular disease | 0.8 | 0.8 | 0.07 | 0.7 | 0.8 | 0.14 |
| Chronic pulmonary disease | 1.5 | 2.6 | 1.11 | 1.8 | 2.6 | 0.87 |
| Prior myocardial infarction | 5.5 | 4.7 | 0.76 | 5.5 | 4.8 | 0.78 |
| Prior PCI | 6.6 | 4.9 | 1.70 | 6.5 | 4.9 | 1.59 |
| Prior major bleeding event | 9.9 | 11.4 | 1.51 | 9.8 | 11.4 | 1.58 |
| Alcohol dependence | 2.9 | 4.1 | 1.20 | 3.2 | 4.1 | 0.96 |
| Hepatic disease | 1.2 | 1.3 | 0.08 | 1.5 | 1.3 | 0.17 |
| History of cancer | 6.6 | 7.7 | 1.10 | 6.6 | 7.6 | 1.05 |
| eGFR ≥ 60 | 94.2 | 92.4 | 1.82 | 93.6 | 92.4 | 1.17 |
| eGFR 45-59 | 3.7 | 4.6 | 0.94 | 4.3 | 4.6 | 0.23 |
| eGFR 30-44 | 0.8 | 1.5 | 0.7 | 0.8 | 1.5 | 0.70 |
| eGFR 15-29 | 0.3 | 0.5 | 0.24 | 0.3 | 0.5 | 0.28 |
| eGFR < 15 | 1.1 | 1 | 0.06 | 1.1 | 1 | 0.04 |
| Preoperative dialysis | 0.8 | 0.8 | 0.01 | 0.8 | 0.8 | 0.01 |
| Pacemaker/ICD | 2.7 | 1.8 | 0.99 | 2.9 | 1.8 | 1.10 |
| Prior heart failure | 16.9 | 15.9 | 1.07 | 17.3 | 15.9 | 1.37 |
| LVEF >50% | 74.8 | 75.1 | 0.23 | 75.7 | 75 | 0.77 |
| LVEF 30-50% | 20.7 | 19.9 | 0.82 | 20 | 20 | 0.02 |
| LVEF <30% | 4.4 | 5 | 0.60 | 4.3 | 5 | 0.79 |
| Prior endocarditis | 2.7 | 2.4 | 0.32 | 2.7 | 2.5 | 0.22 |
| Emergency operation | 1.8 | 3.3 | 1.43 | 2 | 3.2 | 1.21 |
| Isolated AVR | 62.5 | 52.2 | 10.3 | 55.6 | 53.2 | 2.36 |
| Concomitant CABG | 12.2 | 11.4 | 0.78 | 12.5 | 11.5 | 1.01 |
| Ascending aortic surgery | 28.2 | 39.4 | 11.2 | 35.7 | 38.2 | 2.50 |
| DHCA | 3.5 | 8.7 | 5.24 | 6.4 | 8.5 | 2.11 |
| Values are mean (SD) or %. SD, Standard Deviation. Q1-Q4, quartiles 1-4. BMI, body mass index. COPD, chronic obstructive pulmonary disease. PCI, percutaneous coronary intervention. eGFR, estimated glomerular filtration rate. ICD, implantable cardioverter-defibrillator. LVEF, left ventricular ejection fraction. AVR, aortic valve replacement. CABG, coronary artery bypass graft surgery. DHCA, deep hypothermic circulatory arrest. AMD = absolute mean difference (%).  *For continuous variables, the standardized mean difference is shown instead of the AMD. | | | | | | |

| **Supplemental Table S7. Baseline characteristics of patients who received On-X aortic mechanical valve or other mechanical valves in Sweden between 2014 and 2022 after overlap weighting.** | | | | | | |
| --- | --- | --- | --- | --- | --- | --- |
|  | **Before weighting, %** | | | **After weighting, %** | | |
| Variable | On-X aortic valve | Other mechanical valves | Absolute mean difference | On-X aortic valve | Other mechanical valves | Absolute mean difference |
| Age, years (mean [SD]) * | 52.9 (10.6) | 53.6 (10.8) | 0.07 | 53 (10.7) | 53 (10.8) | 0 |
| Female sex | 17.7 | 24.9 | 7.24 | 19.2 | 19.2 | 0 |
| Married | 53.5 | 50.7 | 2.82 | 52.8 | 52.8 | 0 |
| Education <10 years | 13.1 | 16.9 | 3.75 | 13.9 | 13.9 | 0 |
| Education 10-12 years | 56.1 | 51.5 | 4.57 | 54.9 | 54.9 | 0 |
| Education >12 years | 30.8 | 31.6 | 0.83 | 31.2 | 31.2 | 0 |
| Non-Nordic birth region | 5.6 | 12.4 | 6.78 | 6.6 | 6.6 | 0 |
| Household income Q1 (Lowest) | 24.4 | 25.2 | 0.79 | 24.8 | 24.8 | 0 |
| Household income Q2 | 22.3 | 25.8 | 3.51 | 23 | 23 | 0 |
| Household income Q3 | 25.9 | 24.8 | 1.16 | 25.7 | 25.7 | 0 |
| Household income Q4 (Highest) | 27.4 | 24.3 | 3.14 | 26.6 | 26.6 | 0 |
| BMI <18.5 | 0.6 | 0.9 | 0.27 | 0.7 | 0.7 | 0 |
| BMI 18.5-24.9 | 28.8 | 26.7 | 2.09 | 28.4 | 28.4 | 0 |
| BMI 25-29.9 | 39.9 | 40.1 | 0.13 | 39.9 | 39.9 | 0 |
| BMI >30 | 30.6 | 32.3 | 1.69 | 31 | 31 | 0 |
| Diabetes mellitus | 11 | 11.8 | 0.78 | 11.1 | 11.1 | 0 |
| Prior atrial fibrillation | 10.1 | 11.7 | 1.65 | 10.3 | 10.3 | 0 |
| Prior hypertension | 43.9 | 42.7 | 1.20 | 43.6 | 43.6 | 0 |
| Hyperlipidemia | 15.9 | 16.4 | 0.54 | 16 | 16 | 0 |
| Prior stroke | 6.7 | 6.4 | 0.35 | 6.7 | 6.7 | 0 |
| Peripheral vascular disease | 0.8 | 0.8 | 0.07 | 0.8 | 0.8 | 0 |
| Chronic pulmonary disease | 1.5 | 2.6 | 1.11 | 1.7 | 1.7 | 0 |
| Prior myocardial infarction | 5.5 | 4.7 | 0.76 | 5.3 | 5.3 | 0 |
| Prior PCI | 6.6 | 4.9 | 1.70 | 6 | 6 | 0 |
| Prior major bleeding event | 9.9 | 11.4 | 1.51 | 10.2 | 10.2 | 0 |
| Alcohol dependence | 2.9 | 4.1 | 1.20 | 3.2 | 3.2 | 0 |
| Hepatic disease | 1.2 | 1.3 | 0.08 | 1.3 | 1.3 | 0 |
| History of cancer | 6.6 | 7.7 | 1.10 | 6.8 | 6.8 | 0 |
| eGFR ≥ 60 | 94.2 | 92.4 | 1.82 | 93.9 | 93.9 | 0 |
| eGFR 45-59 | 3.7 | 4.6 | 0.94 | 3.8 | 3.8 | 0 |
| eGFR 30-44 | 0.8 | 1.5 | 0.70 | 0.9 | 0.9 | 0 |
| eGFR 15-29 | 0.3 | 0.5 | 0.24 | 0.3 | 0.3 | 0 |
| eGFR < 15 | 1.1 | 1 | 0.06 | 1.1 | 1.1 | 0 |
| Preoperative dialysis | 0.8 | 0.8 | 0.01 | 0.8 | 0.8 | 0 |
| Pacemaker/ICD | 2.7 | 1.8 | 0.99 | 2.5 | 2.5 | 0 |
| Prior heart failure | 16.9 | 15.9 | 1.07 | 16.7 | 16.7 | 0 |
| LVEF >50% | 74.8 | 75.1 | 0.23 | 74.8 | 74.8 | 0 |
| LVEF 30-50% | 20.7 | 19.9 | 0.82 | 20.6 | 20.6 | 0 |
| LVEF <30% | 4.4 | 5 | 0.60 | 4.5 | 4.5 | 0 |
| Prior endocarditis | 2.7 | 2.4 | 0.32 | 2.7 | 2.7 | 0 |
| Emergency operation | 1.8 | 3.3 | 1.43 | 2 | 2 | 0 |
| Isolated AVR | 62.3 | 52.2 | 10.15 | 60 | 60 | 0 |
| Concomitant CABG | 12.2 | 11.4 | 0.78 | 12.2 | 12.2 | 0 |
| Ascending aortic surgery | 28.2 | 39.4 | 11.2 | 30.8 | 30.8 | 0 |
| DHCA | 3.5 | 8.7 | 5.24 | 4.2 | 4.2 | 0 |
| Values are mean (SD) or %. SD, Standard Deviation. Q1-Q4, quartiles 1-4. BMI, body mass index. COPD, chronic obstructive pulmonary disease. PCI, percutaneous coronary intervention. eGFR, estimated glomerular filtration rate. ICD, implantable cardioverter-defibrillator. LVEF, left ventricular ejection fraction. AVR, aortic valve replacement. CABG, coronary artery bypass graft surgery. DHCA, deep hypothermic circulatory arrest. AMD = absolute mean difference (%).  *For continuous variables, the standardized mean difference is shown instead of the AMD. | | | | | | |

| **Supplemental Table S8.** Cumulative incidence of clinical outcomes in the weighted population | | |
| --- | --- | --- |
|  | **On-X aortic valve** | **Other mechanical valves** |
| **Major bleeding events** | | |
| 4 years | 4.0 (2.6-6.2) | 3.2 (2.5-4.1) |
| 8 years | 7.2 (4.8-11) | 7.0 (5.5-8.8) |
| **Thromboembolic events** | | |
| 4 years | 5.1 (3.5-7.4) | 4.8 (4.0-5.8) |
| 8 years | 7.3 (5.3-10.2) | 6.4 (5.0-7.9) |
| **All-cause mortality** | | |
| 4 years | 3.6 (2.3-5.7) | 4.6 (4-5.7) |
| 8 years | 7.0 (4.6-11) | 9.6 (8-12) |
| **Aortic valve reintervention** | | |
| 4 years | 1.2 (0.6-2.4) | 2.1 (1.0-2.9) |
| 8 years | 4.7 (1.7-13) | 3.2 (2.0-4.6) |
| The number are % (95% CI). CI = confidence interval. Adjusted by optimization-based weights. The competing risk of death was accounted for in the outcomes major bleeding events, thromboembolic events and aortic valve reintervention. | | |

| **Supplemental Table S9. Crude cumulative incidence for major bleeding events, thromboembolic events, all-cause mortality, and aortic valve reintervention. % (95% CI)** | | |
| --- | --- | --- |
| **Outcome** | **4 years** | **8 years** |
| **Major bleeding events** | | |
| On-X aortic valve | 3.9 (2.3-5.5) | 7.0 (4.3-9.7) |
| Other mechanical valves | 3.2 (2.4-4.0) | 7.0 (5.4-8.6) |
| **Thromboembolic events** | | |
| On-X aortic valve | 5.2 (3.4-7.1) | 7.8 (5.3-10.3) |
| Other mechanical valves | 4.8 (3.8-5.7) | 6.4 (5.0-7.7) |
| **All-cause mortality** | | |
| On-X aortic valve | 3.7 (2.1-5.3) | 6.9 (4.1-9.7) |
| Other mechanical valves | 4.4 (3.5-5.4) | 9.6 (7.7-11.6) |
| **Reintervention** | | |
| On-X aortic valve | 1.2 (0.4-2.1) | 4.1 (0.1-8.0) |
| Other mechanical valves | 2.1 (1.4-2.8) | 3.3 (2.1-4.4) |
| CI = confidence interval. Major bleeding events, thromboembolic events and aortic valve reintervention using Aalen-Johansen estimator accounting for the competing risk of death. | | |

| **Supplemental Table S10.** Median follow-up time in patients who underwent mechanical aortic valve replacement in Sweden between 2014 and 2022, by group. | |
| --- | --- |
| **Outcome** | **Median follow-up time (Years)** |
| **Major bleeding events** |  |
| On-X aortic valve | 4.30 |
| Other mechanical valves | 4.07 |
| **Thromboembolic events** |  |
| On-X aortic valve | 4.32 |
| Other mechanical valves | 4.07 |
| **All-cause mortality** |  |
| On-X aortic valve | 4.49 |
| Other mechanical valves | 4.28 |
| **Aortic valve reintervention** |  |
| On-X aortic valve | 4.29 |
| Other mechanical valves | 4.09 |
| Derived using the reverse Kaplan-Meier based method.^5^ | |

| **Supplemental Table S11. Frequencies of bleeding events in the study population.** | | | |
| --- | --- | --- | --- |
| **Bleeding frequency** | **On-X aortic valve** | **Other mechanical valves** | **Total** |
| **1** | 22 | 71 | 93 |
| **2** | 2 | 14 | 16 |
| **3** | 2 | 4 | 6 |
| **4** | 2 | 3 | 5 |
| **5** | 1 |  | 1 |
| **6** | 1 |  | 1 |
| Number of patients who receive the On-X aortic valve or other mechanical aortic valves who experienced first- and multiple-time bleeding events in the study population. | | | |

| **Supplemental Table S12. Number of (first time) events between 2014 and 2022.** | | | |
| --- | --- | --- | --- |
| Outcome | On-X aortic valve | Other mechanical | Total events |
| Bleeding | 30 | 92 | 122 |
| Thromboembolic events | 40 | 110 | 150 |
| Reintervention | 10 | 45 | 55 |
| Dead | 29 | 126 | 155 |
| Number of patients who receive the On-X aortic valve or other mechanical aortic valves who experienced first-time event in the study population. | | | |

**Supplemental Figure S1:** Flowchart of study population. SWEDEHEART, Swedish Web-System for Enhancement and Development of Evidence-Based Care in Heart Disease Evaluated According to Recommended Therapies; SAVR, surgical aortic valve replacement; CABG, coronary artery bypass grafting.

**Supplemental Figure S2:** Proportion of On-X aortic valves operated per year in Sweden between 2014 and 2022.


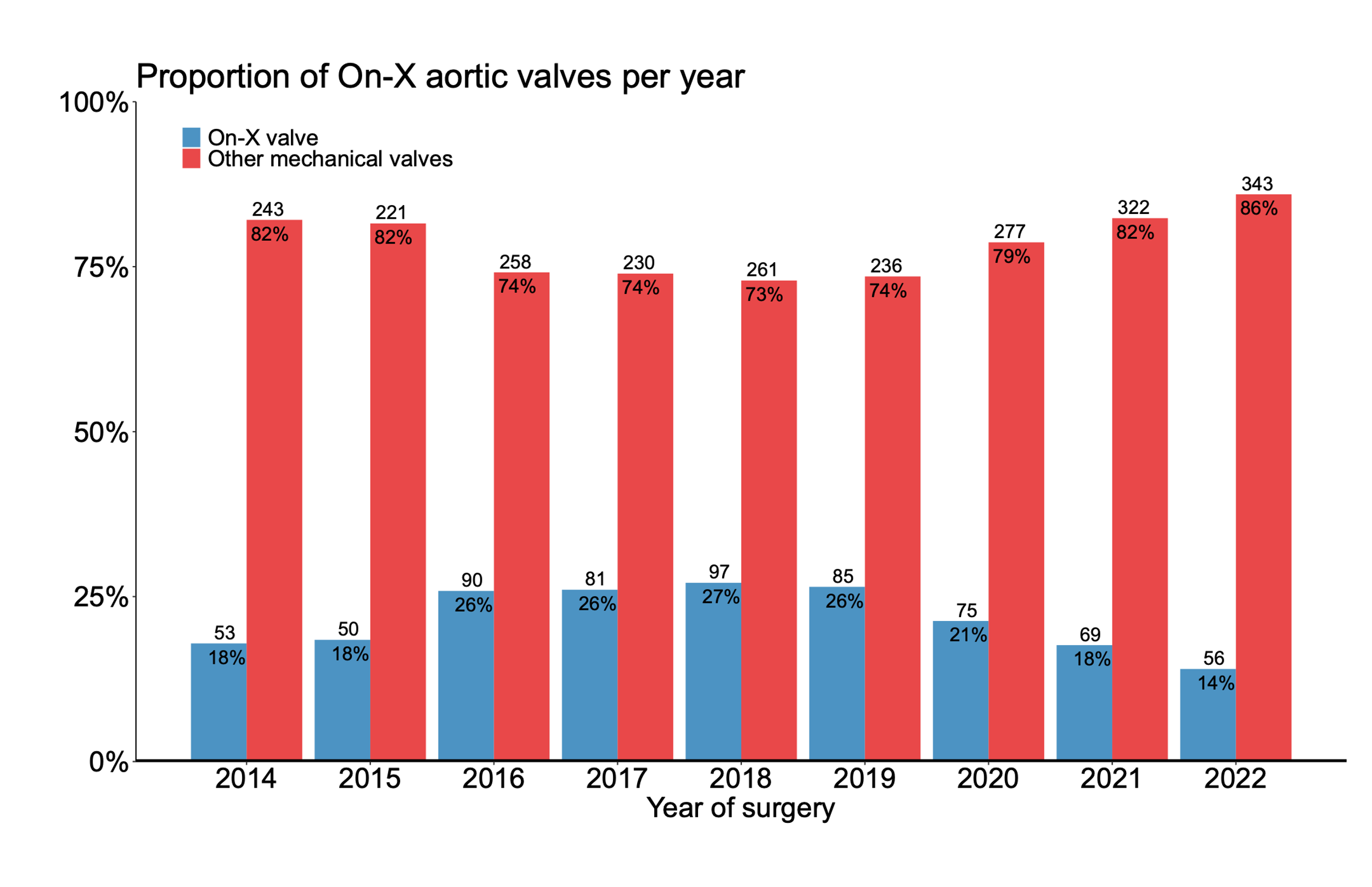


**Supplemental Figure S3:** Absolute mean differences before (red circles) and after (blue circles) optimization-based weighting.


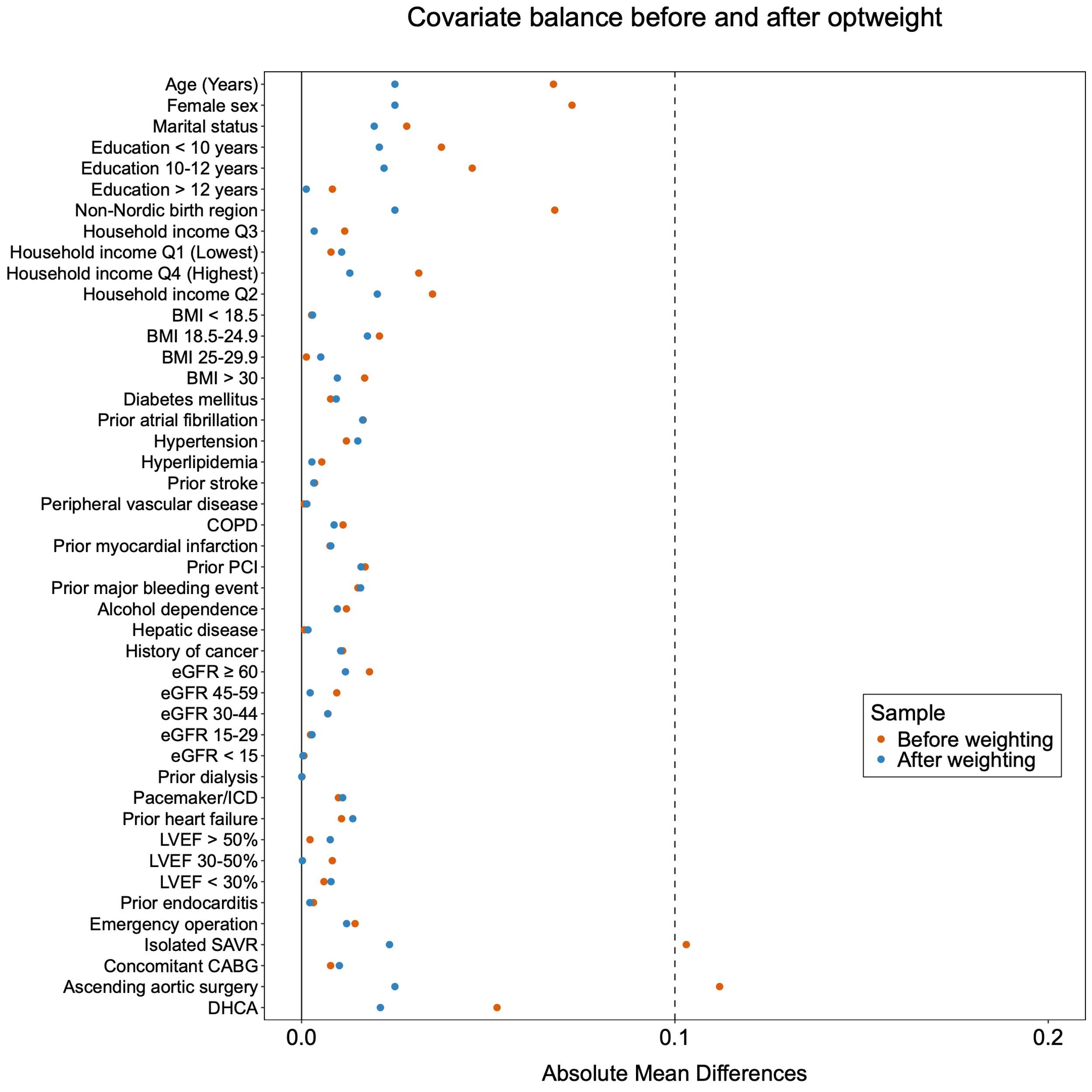


**Supplemental Figure S4:** Absolute mean differences before (red circles) and after (blue circles) overlap weighting.


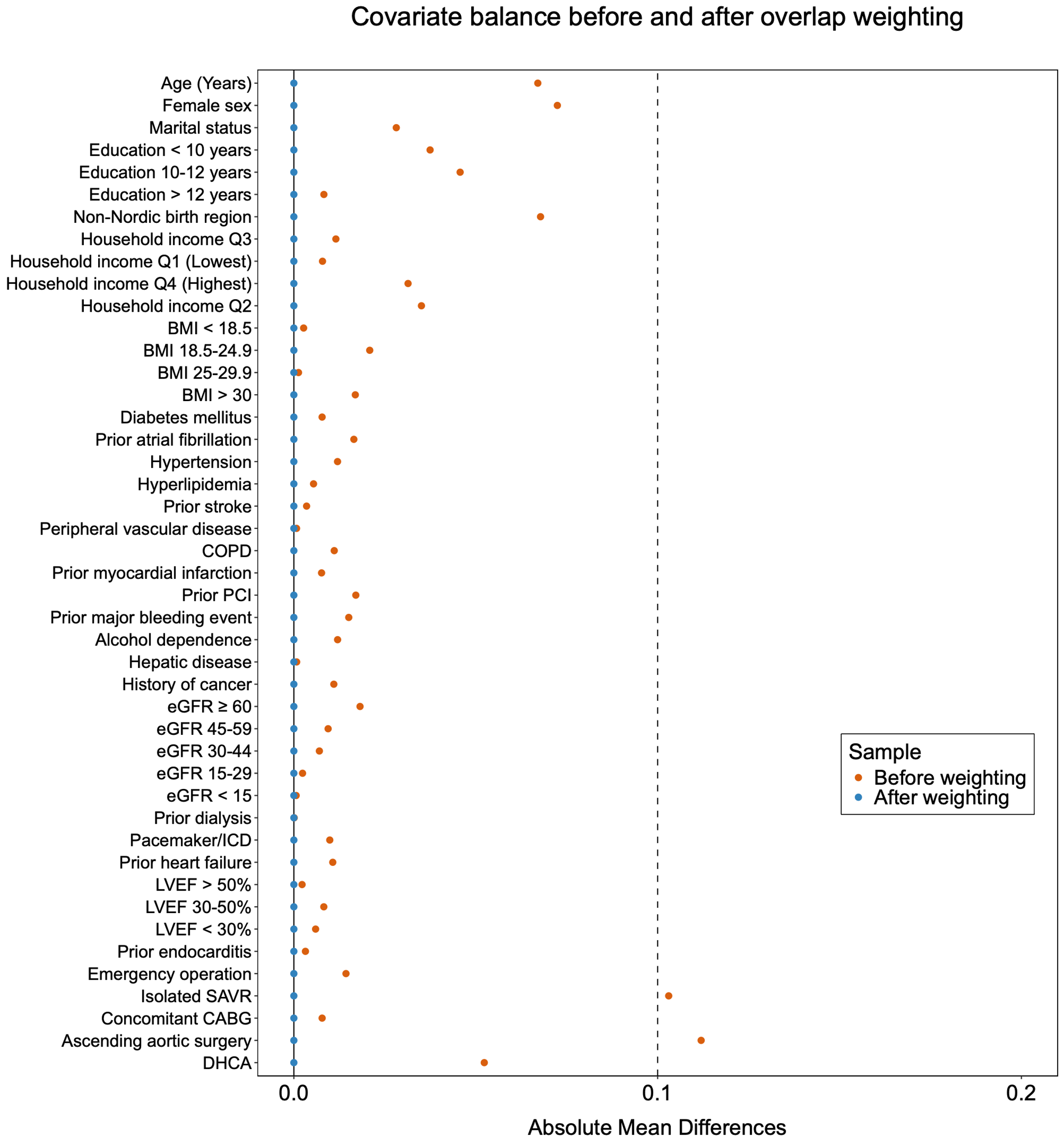


**Supplemental Figure S5:** Aalen-Johansen estimated crude cumulative incidence of major bleeding events in patients who either received an On-X aortic valve or other mechanical valves after surgical AVR in Sweden between 2014 and 2022. Shaded areas represent 95% confidence intervals.


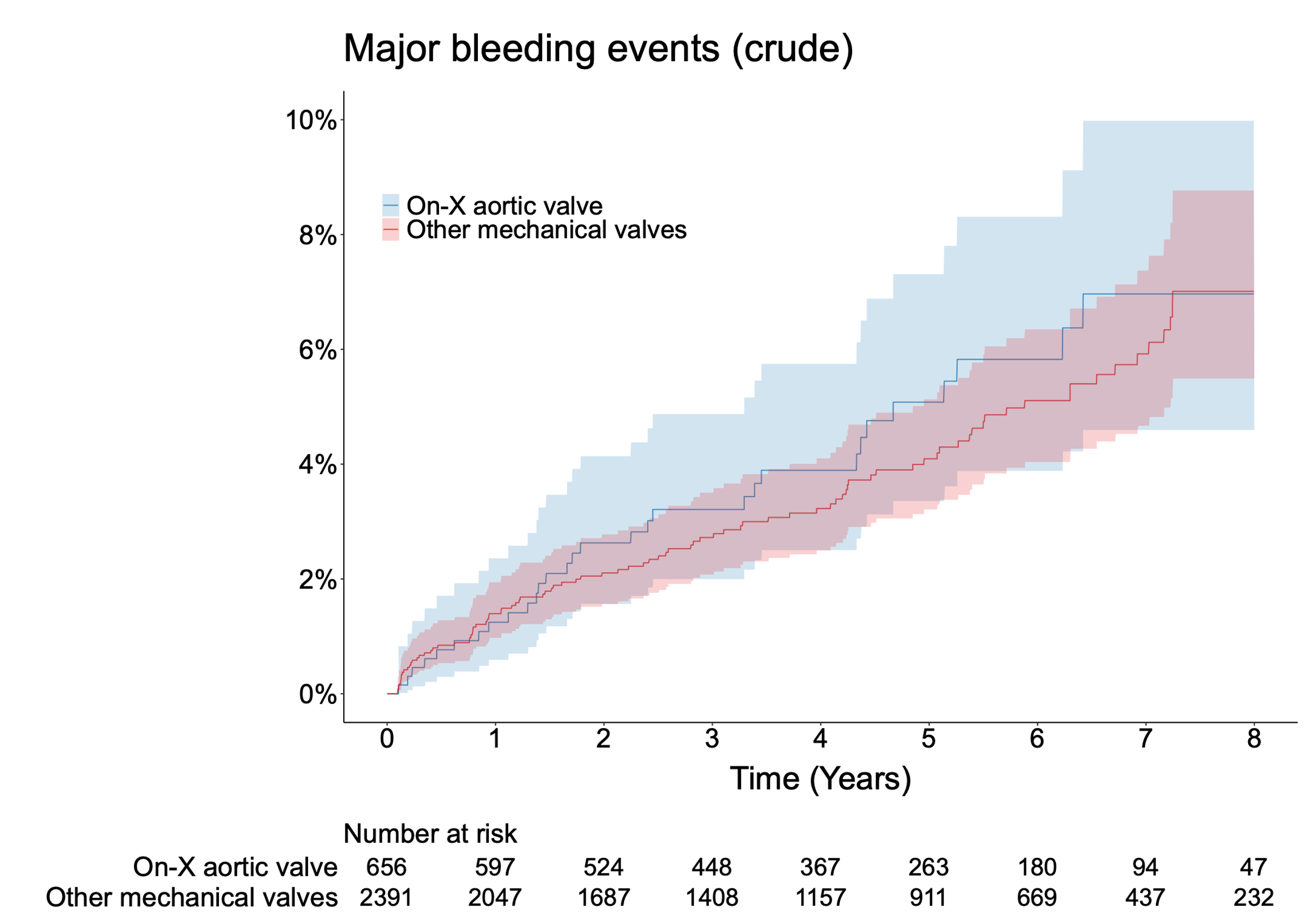


**Supplemental Figure S6:** Aalen-Johansen estimated crude cumulative incidence of thromboembolic events in patients who either received an On-X aortic valve or other mechanical valves after surgical AVR in Sweden between 2014 and 2022. Shaded areas represent 95% confidence intervals.


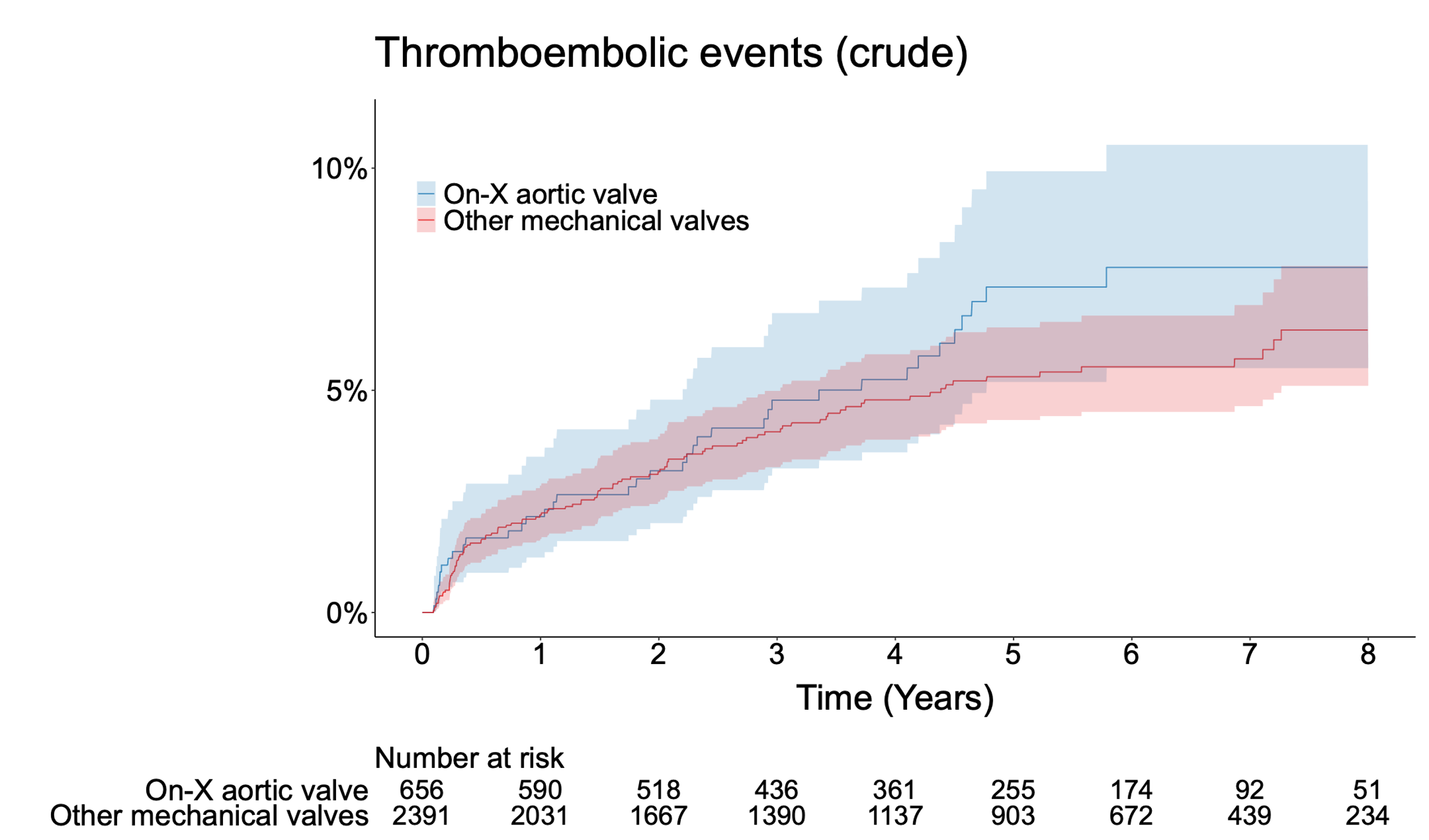


**Supplemental Figure S7:** Kaplan-Meier estimated crude survival in patients who either received an On-X aortic valve or other mechanical valves after surgical AVR in Sweden between 2014 and 2022.


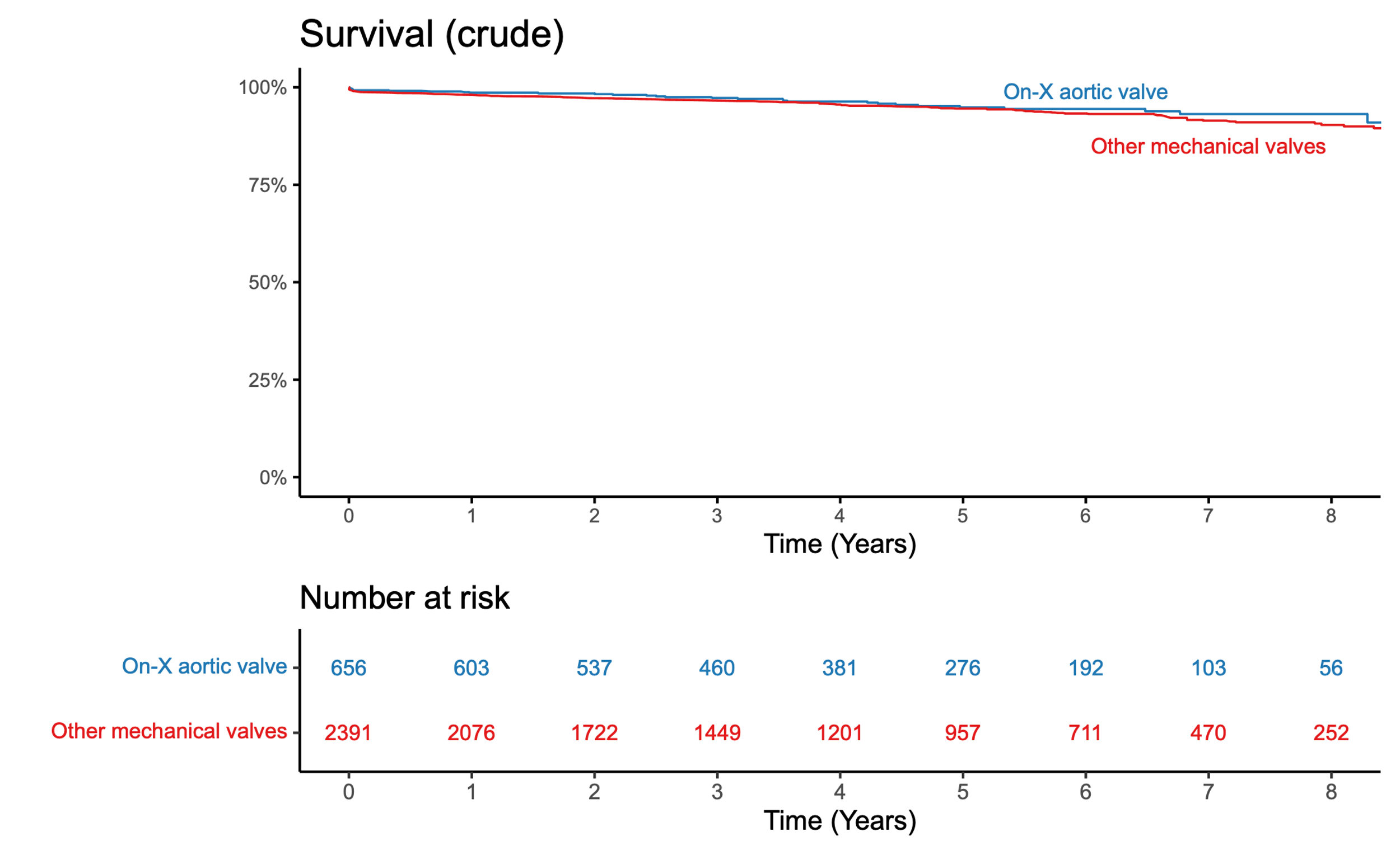


**Supplemental Figure S8:** Aalen-Johansen estimated crude cumulative incidence of aortic valve reintervention in patients who either received an On-X aortic valve or other mechanical valves after surgical AVR in Sweden between 2014 and 2022. Shaded areas represent 95% confidence intervals.


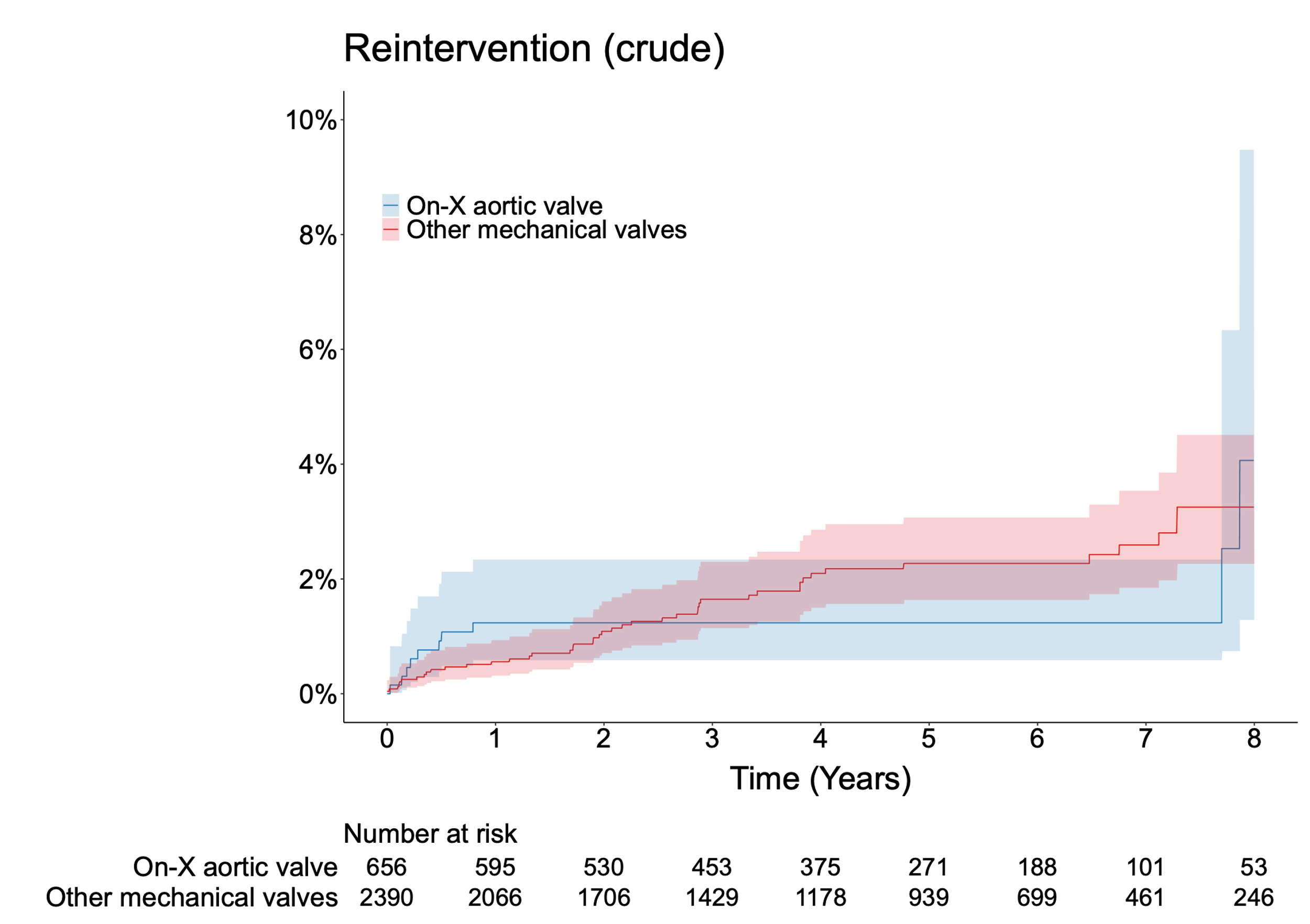


**Supplemental Figure S9:** Cumulative incidence of survival after optimization-based weighting in patients who either received an On-X aortic valve or other mechanical valves after surgical aortic valve replacement in Sweden between 2014 and 2022.

**
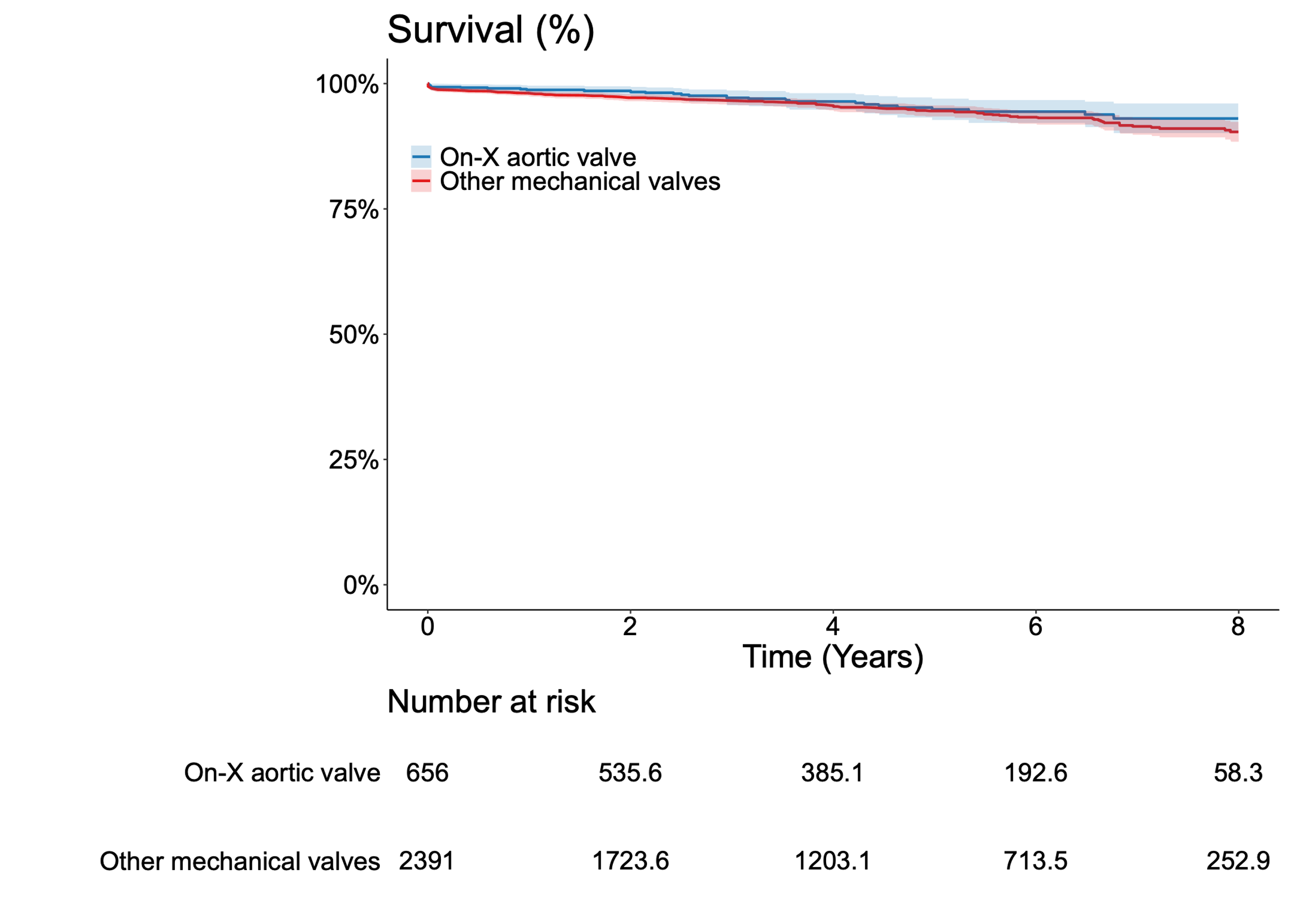
**

**Supplemental Figure S10:** Cumulative incidence of aortic valve reintervention after optimization-based weighting in patients who either received an On-X aortic valve or other mechanical valves after surgical aortic valve replacement in Sweden between 2014 and 2022.

**
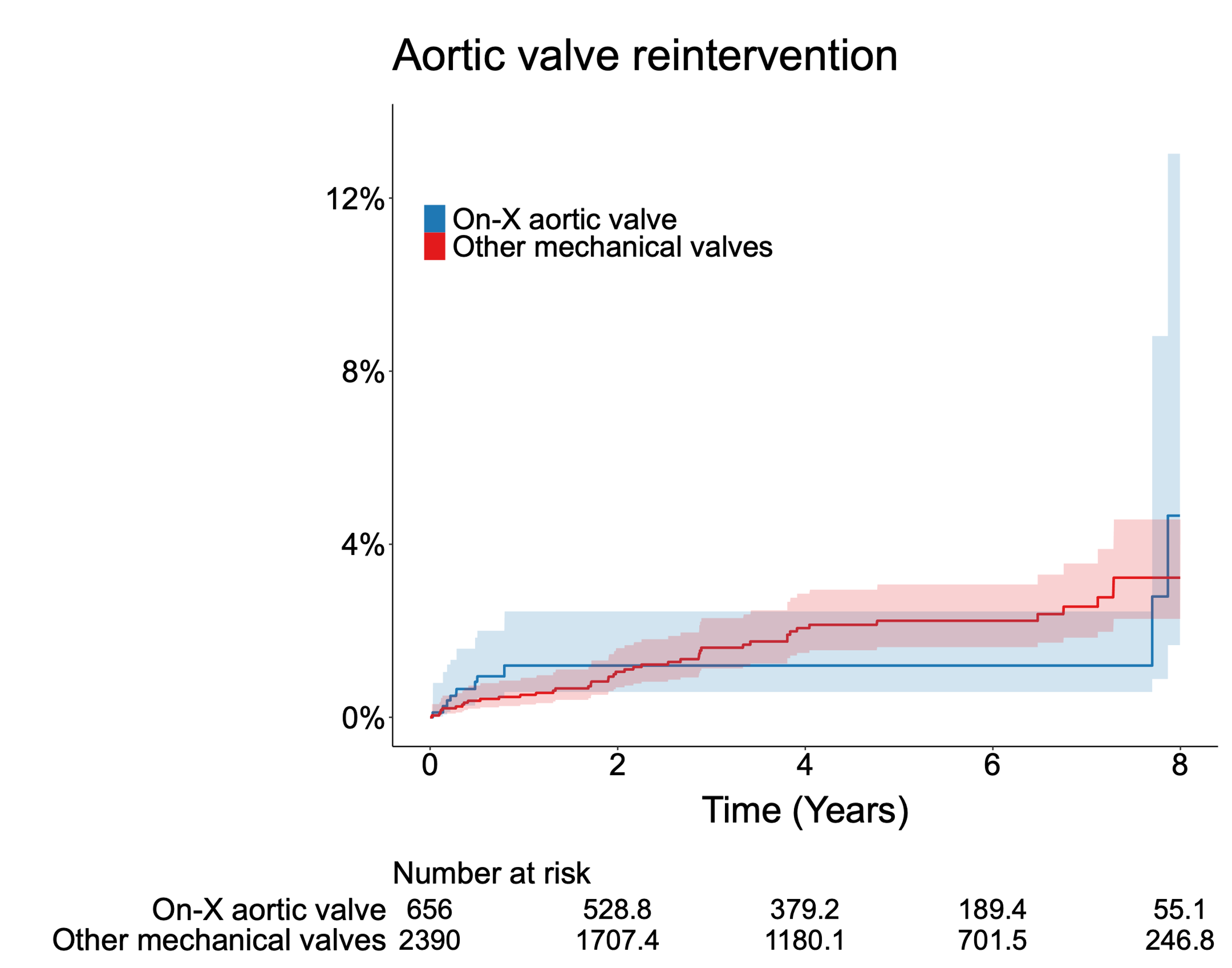
**

**Supplemental Figure S11:** Cumulative incidence of major bleeding events after overlap weighting in patients who either received an On-X aortic valve or other mechanical valves after surgical AVR in Sweden between 2014 and 2022.


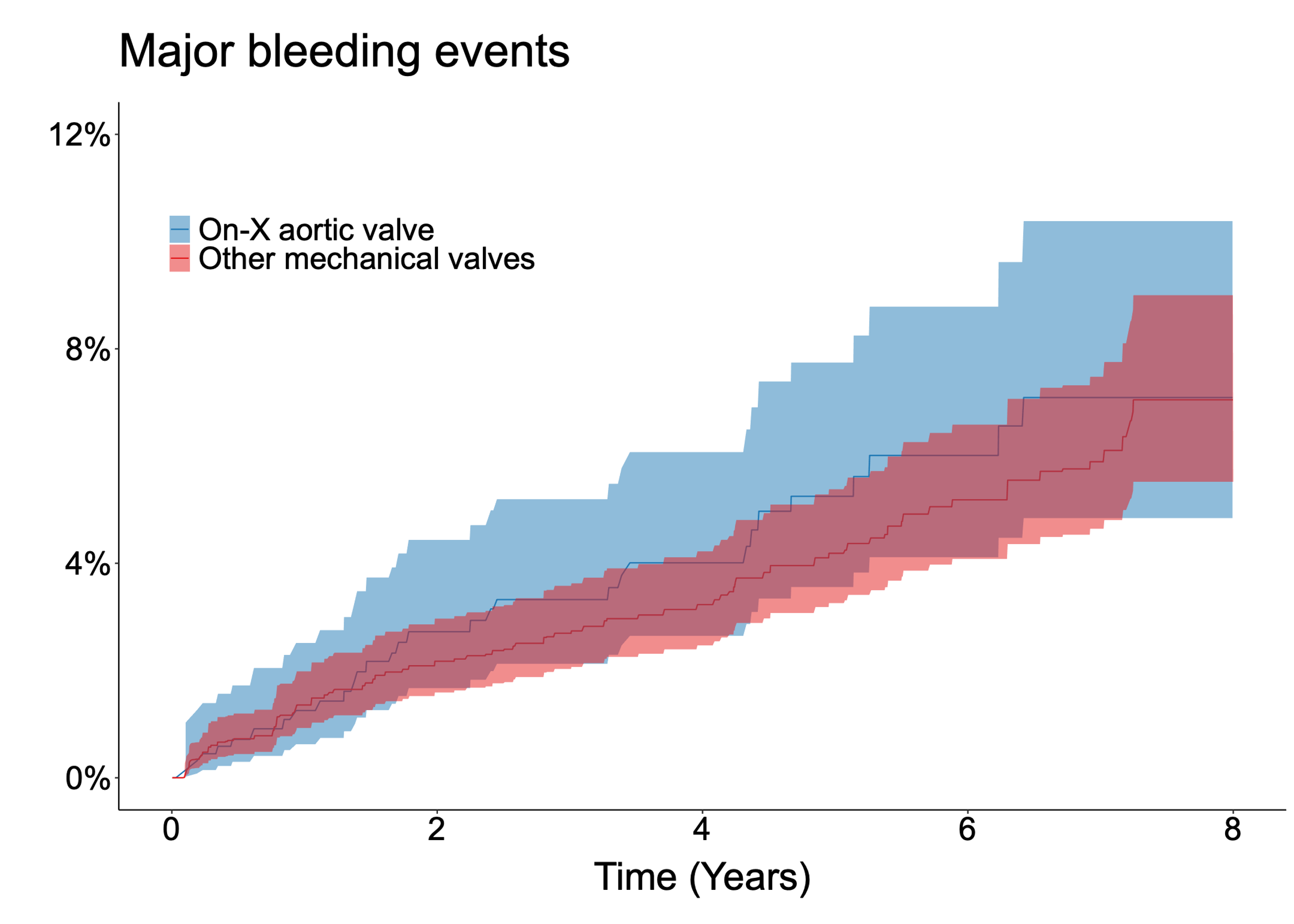


**Supplemental Figure S12:** Cumulative incidence of thromboembolic events after overlap weighting in patients who either received an On-X aortic valve or other mechanical valves after surgical AVR in Sweden between 2014 and 2022.


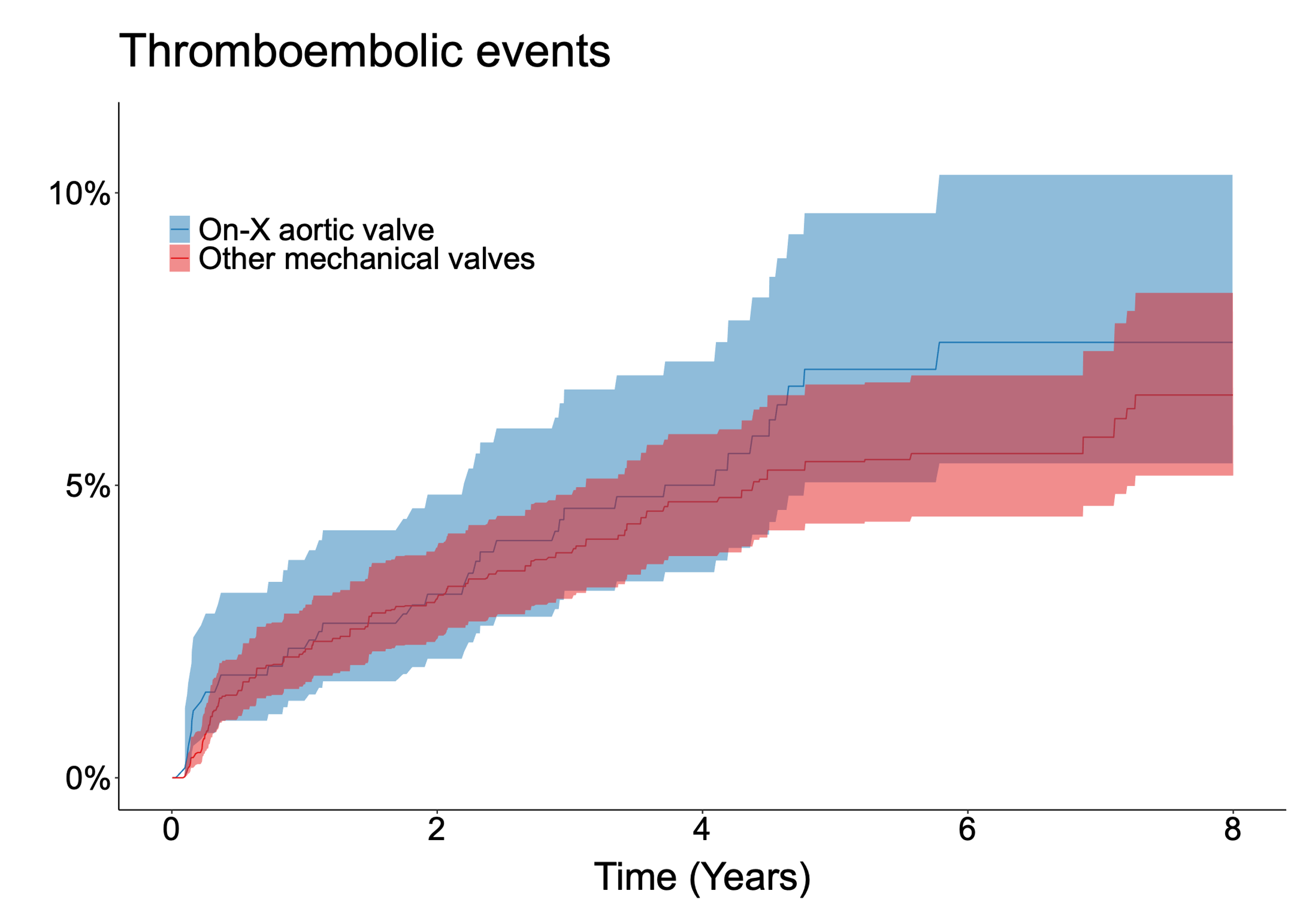


**Supplemental Figure S13:** Cumulative incidence of survival after overlap weighting in patients who either received an On-X aortic valve or other mechanical valves after surgical AVR in Sweden between 2014 and 2022.

**
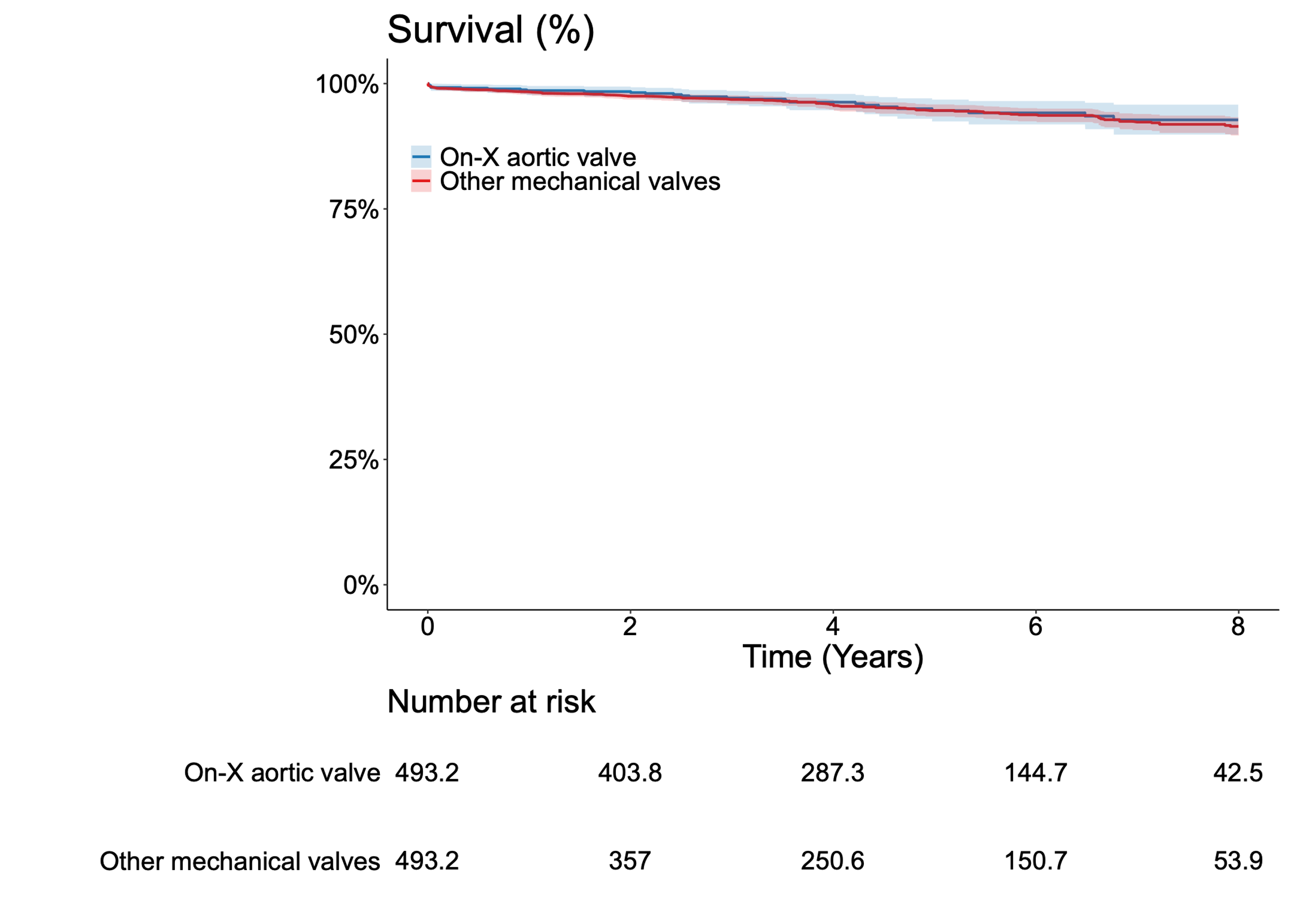
**

**Supplemental Figure S14:** Cumulative incidence of aortic valve reintervention after overlap weighting in patients who either received an On-X aortic valve or other mechanical valves after surgical AVR in Sweden between 2014 and 2022.


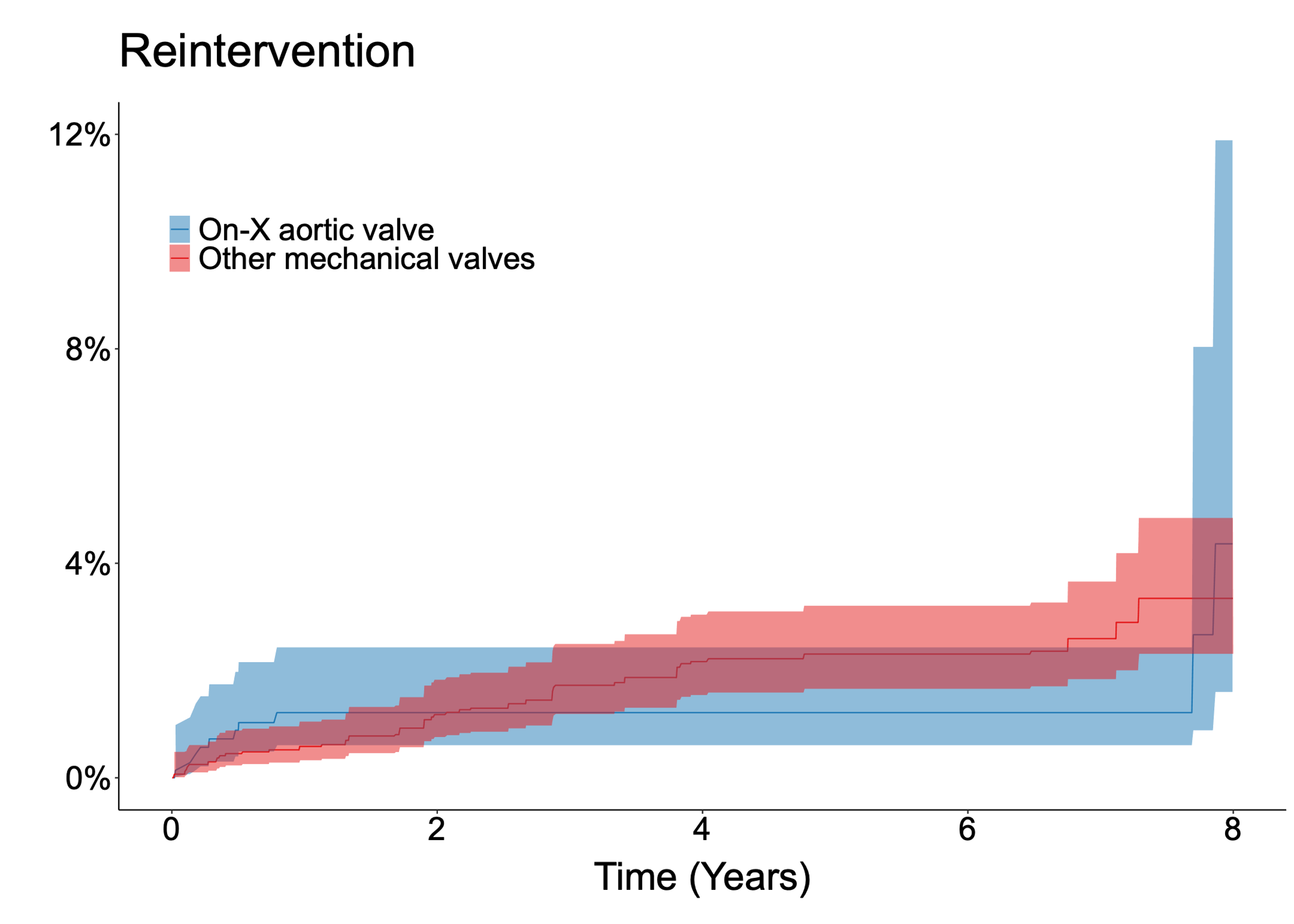


**Supplemental Figure S15:** Absolute mean differences before (red circles) and after (blue circles) optimization-based weighting in patients without preoperative atrial fibrillation.


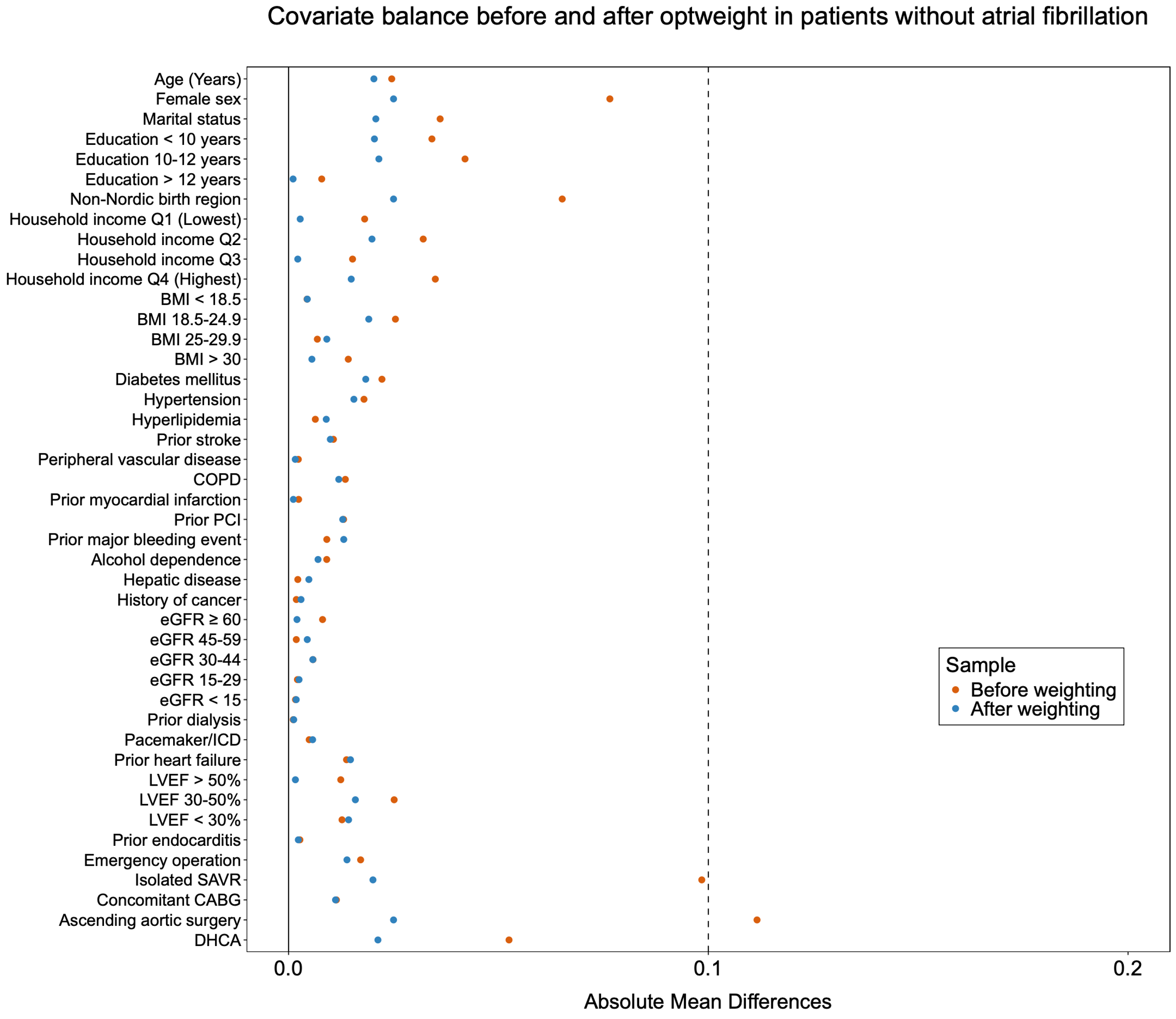


**Supplemental Figure S16:** Cumulative incidence of survival after optimization-based weighting in patients without preoperative atrial fibrillation who either received an On-X aortic valve or other mechanical valves after surgical AVR in Sweden between 2014 and 2022.


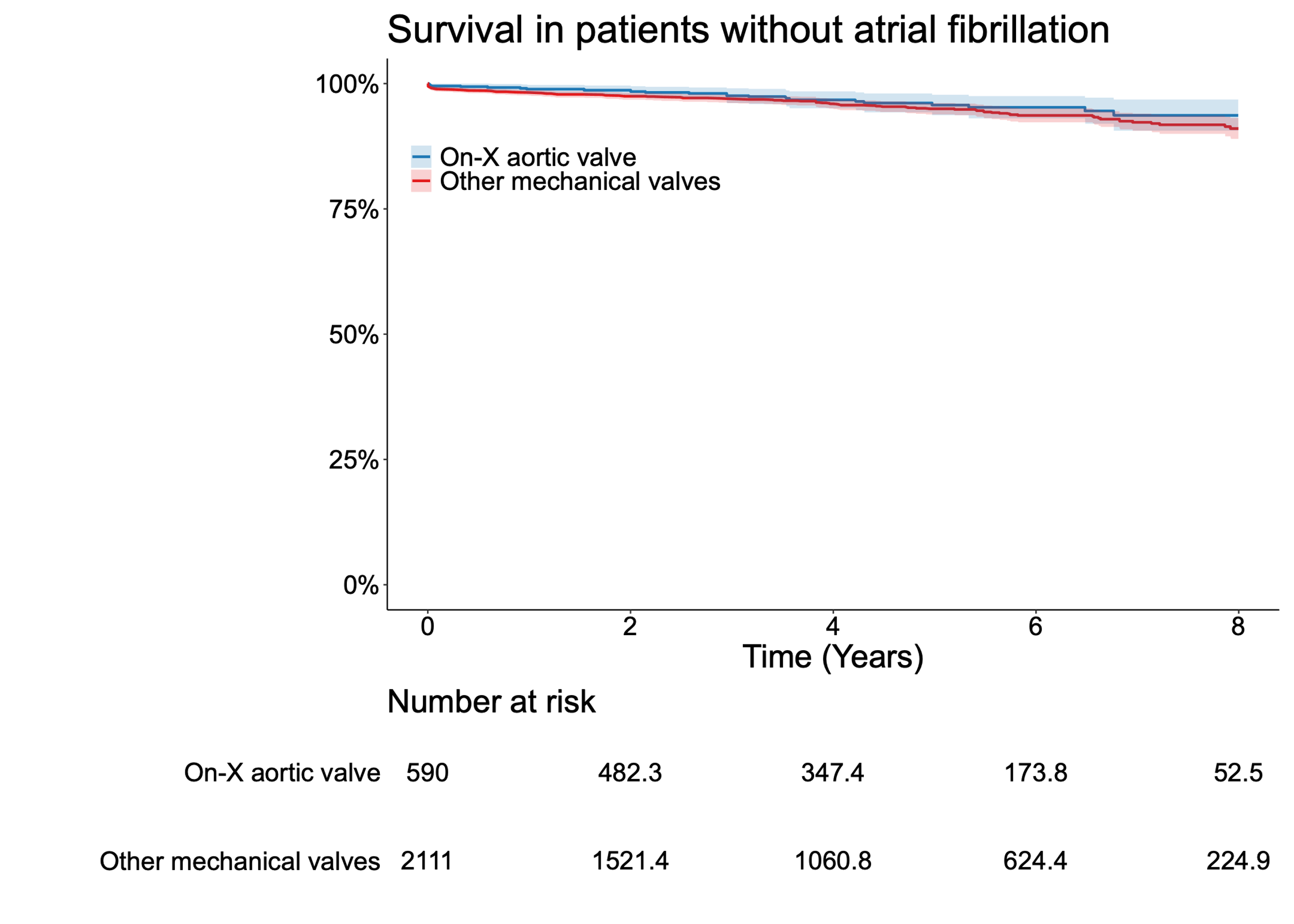


**Supplemental Figure S17:** Cumulative incidence of major bleeding events and thromboembolic events after optimization-based weighting in patients without preoperative atrial fibrillation who either received an On-X aortic valve or other mechanical valves after surgical AVR in Sweden between 2014 and 2022.


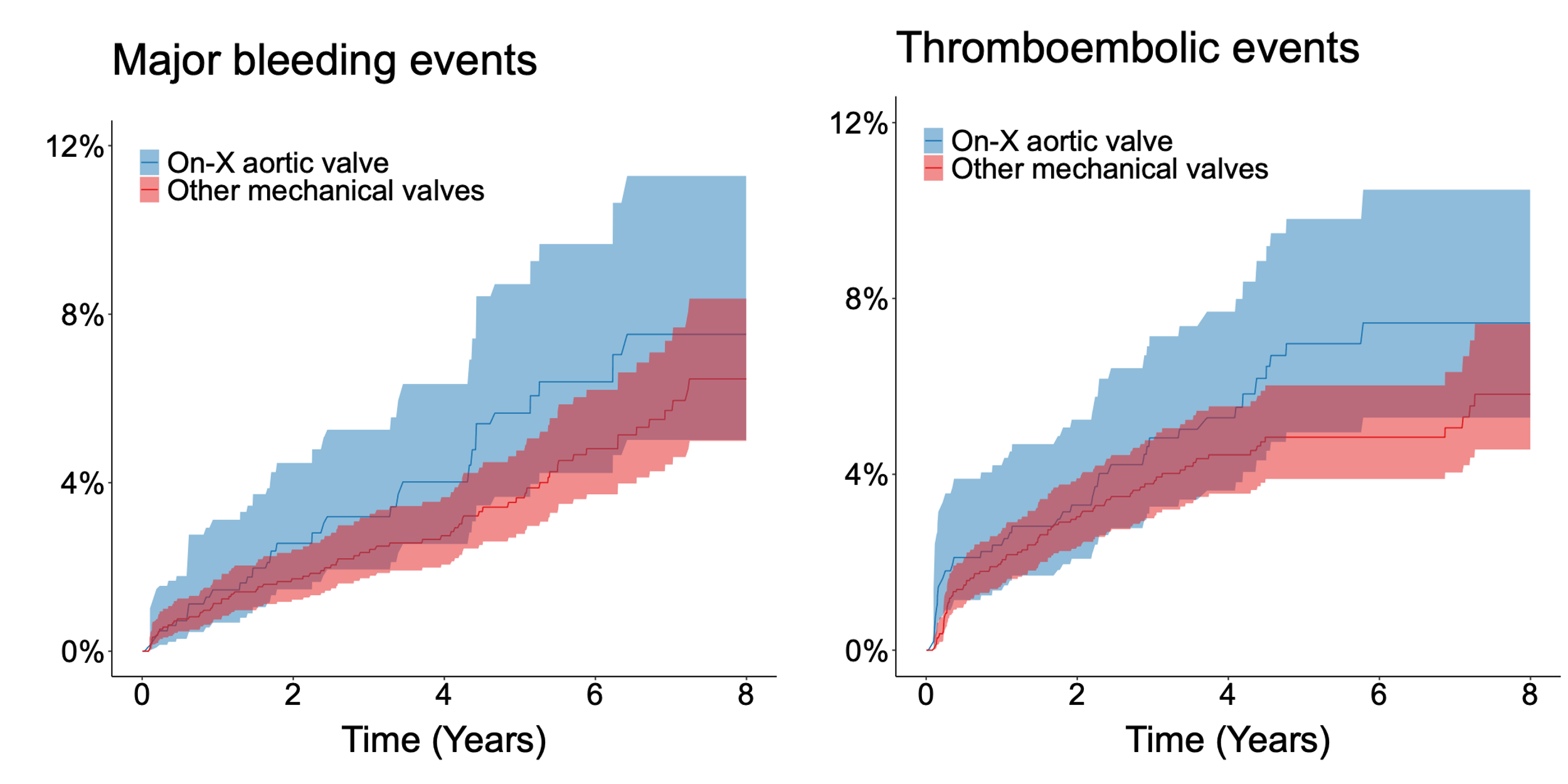


**Supplemental Figure S18:** Absolute mean differences before (red circles) and after (blue circles) optimization-based weighting in patients without preoperative major bleeding events.


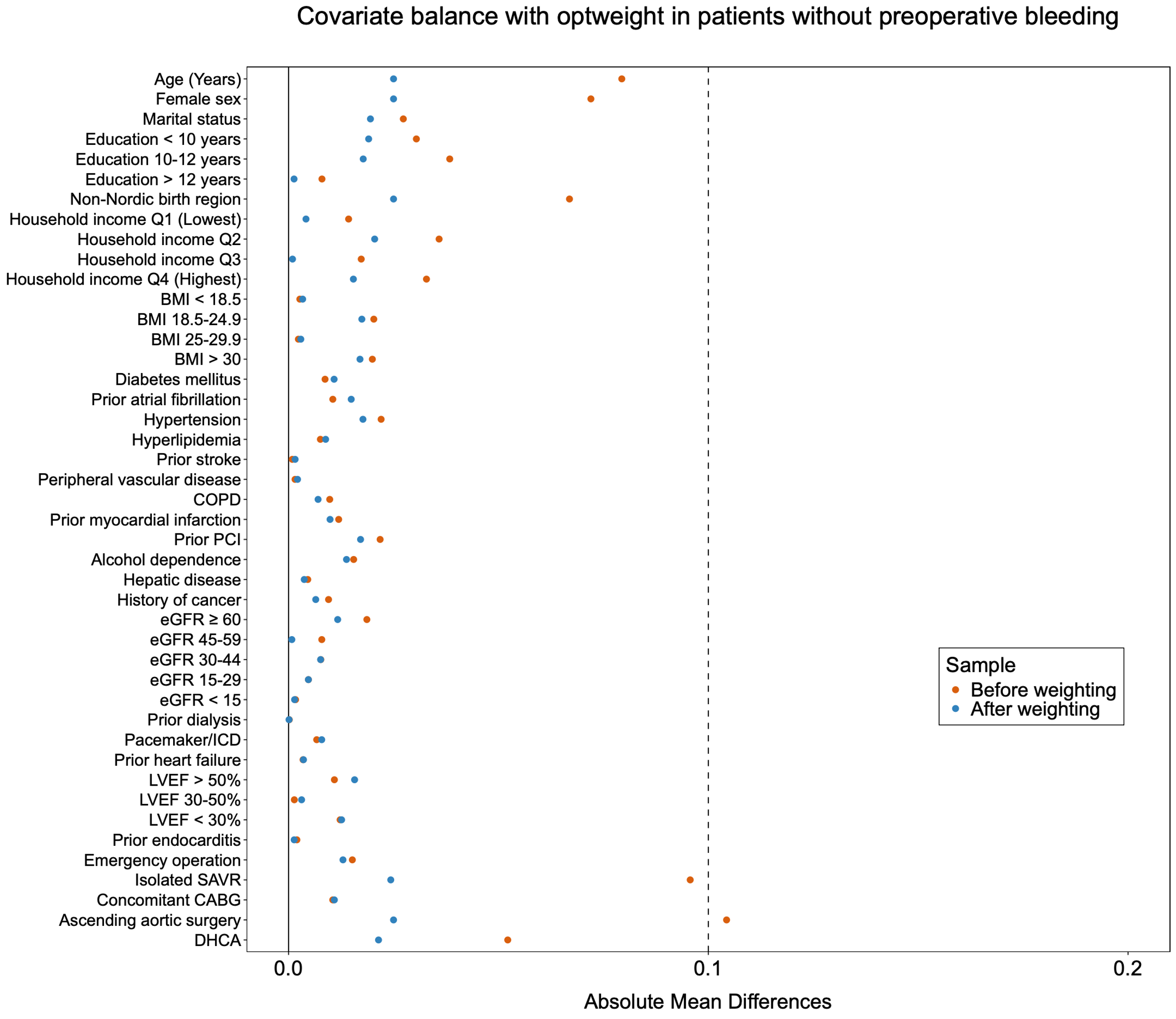


**Supplemental Figure S19:** Cumulative incidence of survival after optimization-based weighting in patients without preoperative major bleeding events who either received an On-X aortic valve or other mechanical valves after surgical AVR in Sweden between 2014 and 2022.


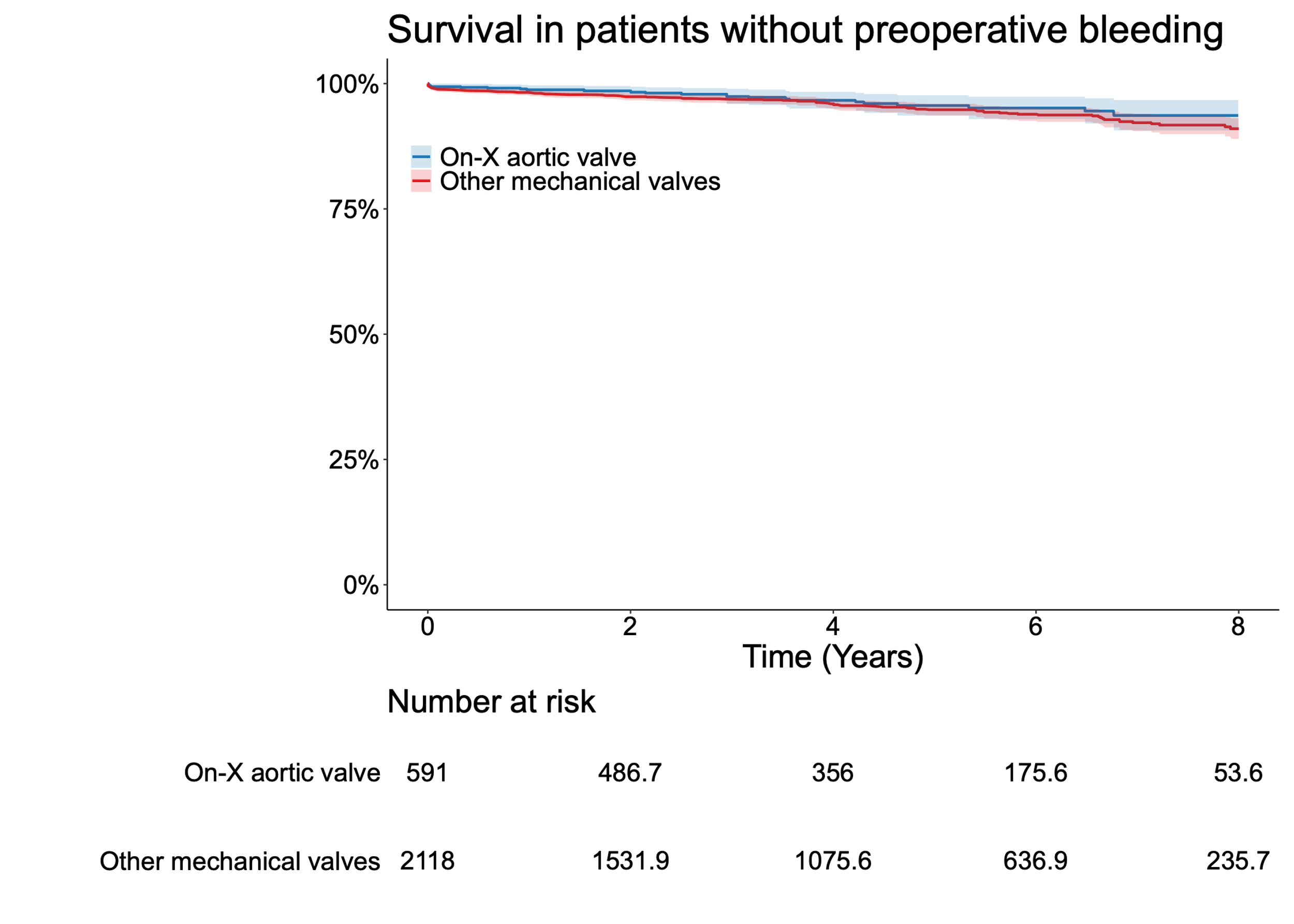


**Supplemental Figure S20:** Cumulative incidence of major bleeding events and thromboembolic events after optimization-based weighting in patients without preoperative major bleeding events who either received an On-X aortic valve or other mechanical valves after surgical AVR in Sweden between 2014 and 2022.


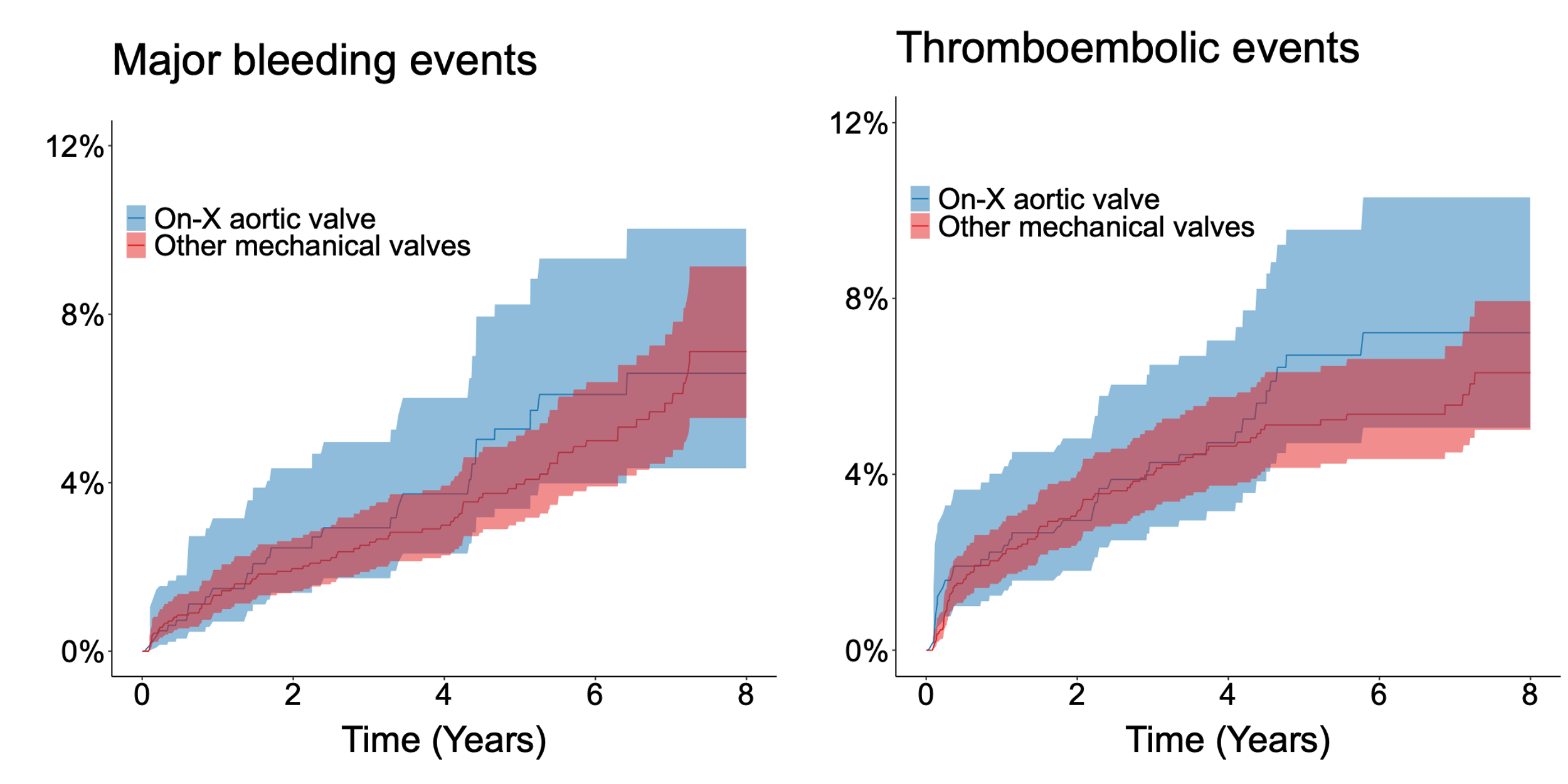


**Supplemental Figure S21:** Absolute mean differences before (red circles) and after (blue circles) optimization-based weighting in patients with underwent isolated AVR.


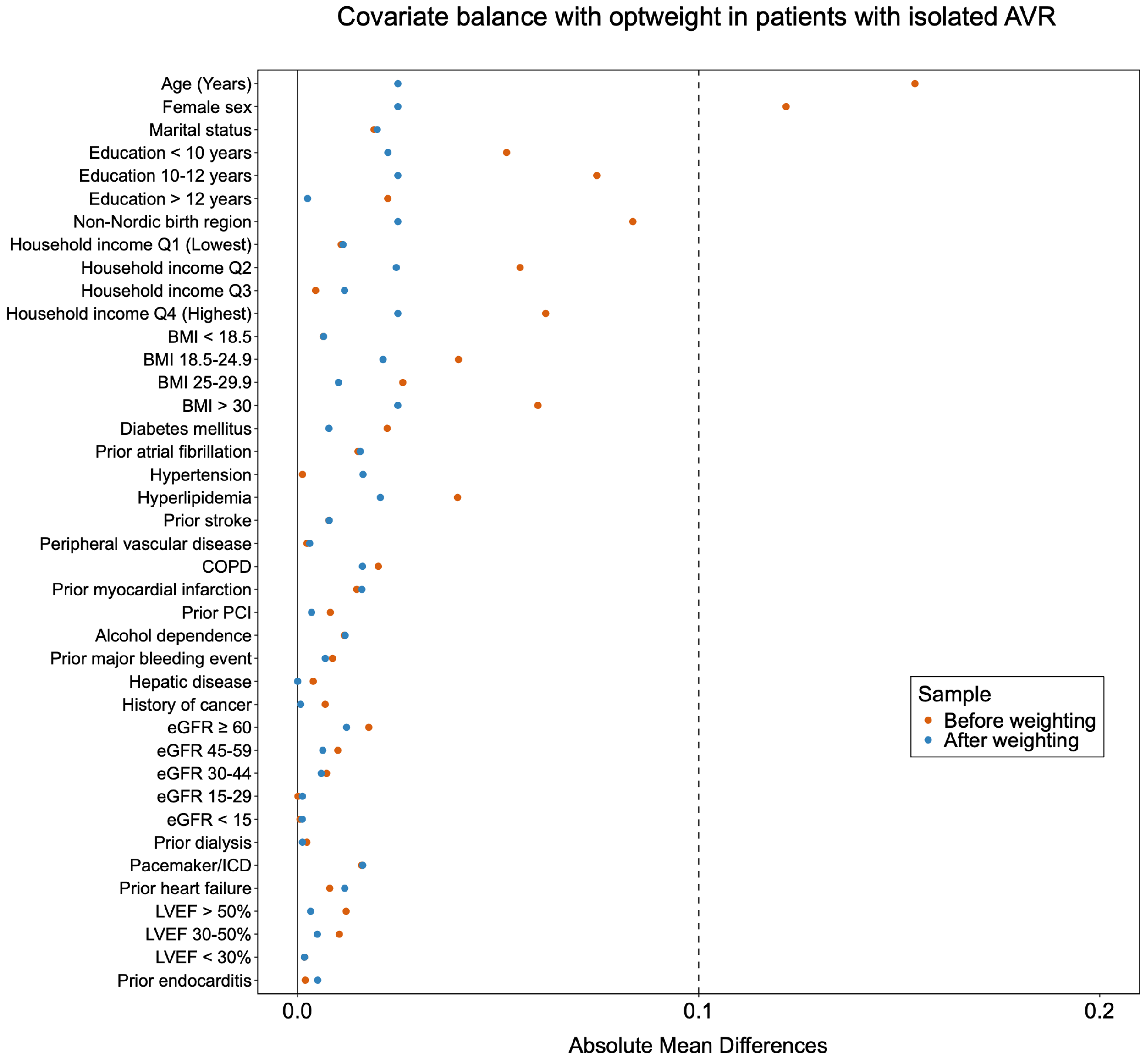


**Supplemental Figure S22:** Cumulative incidence of survival after optimization-based weighting in patients who underwent isolated AVR in Sweden between 2014 and 2022.


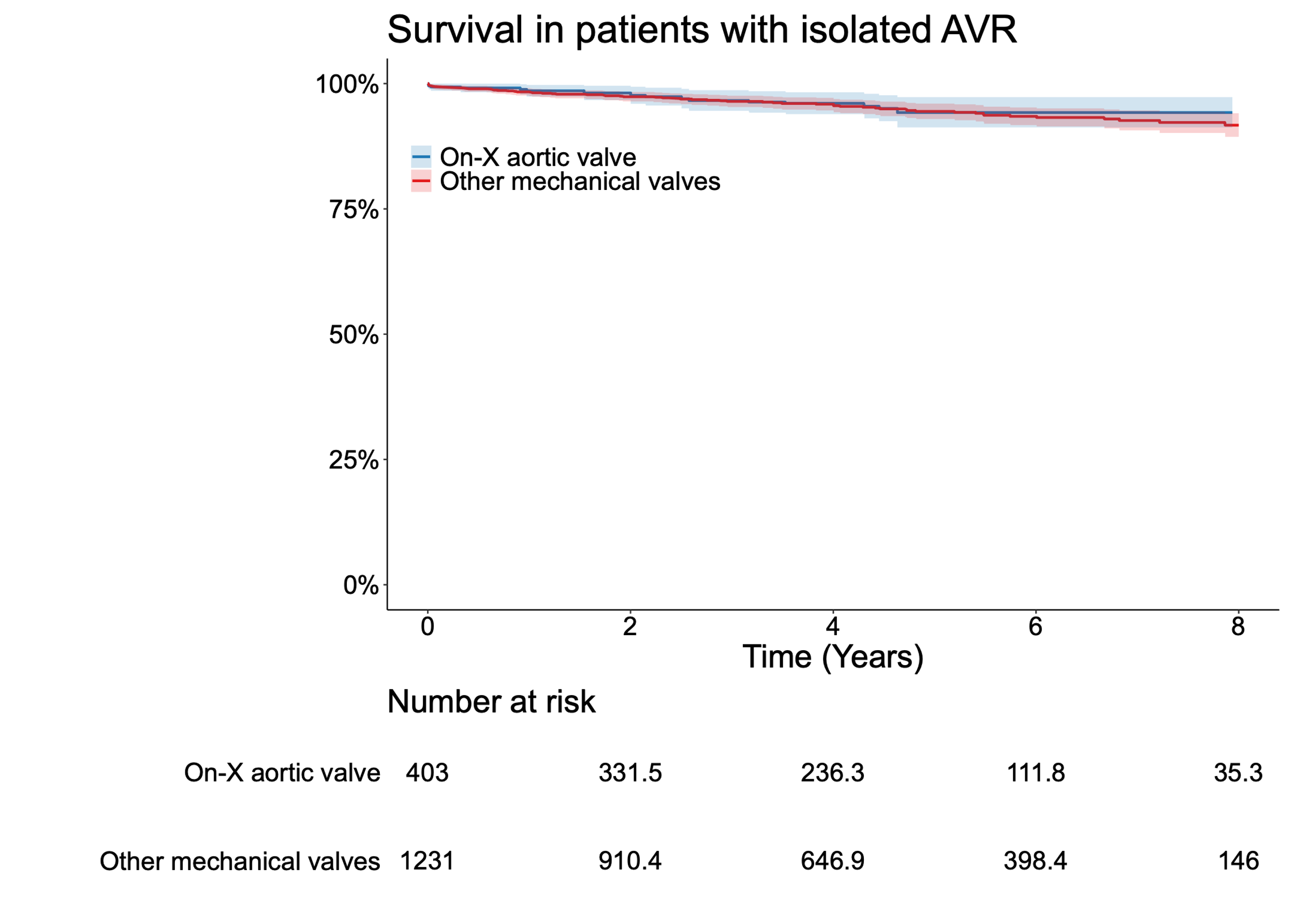


**Supplemental Figure S23:** Cumulative incidence of major bleeding events and thromboembolic events after optimization-based weighting in patients without preoperative major bleeding events who either received an On-X aortic valve or other mechanical valves after surgical AVR in Sweden between 2014 and 2022.


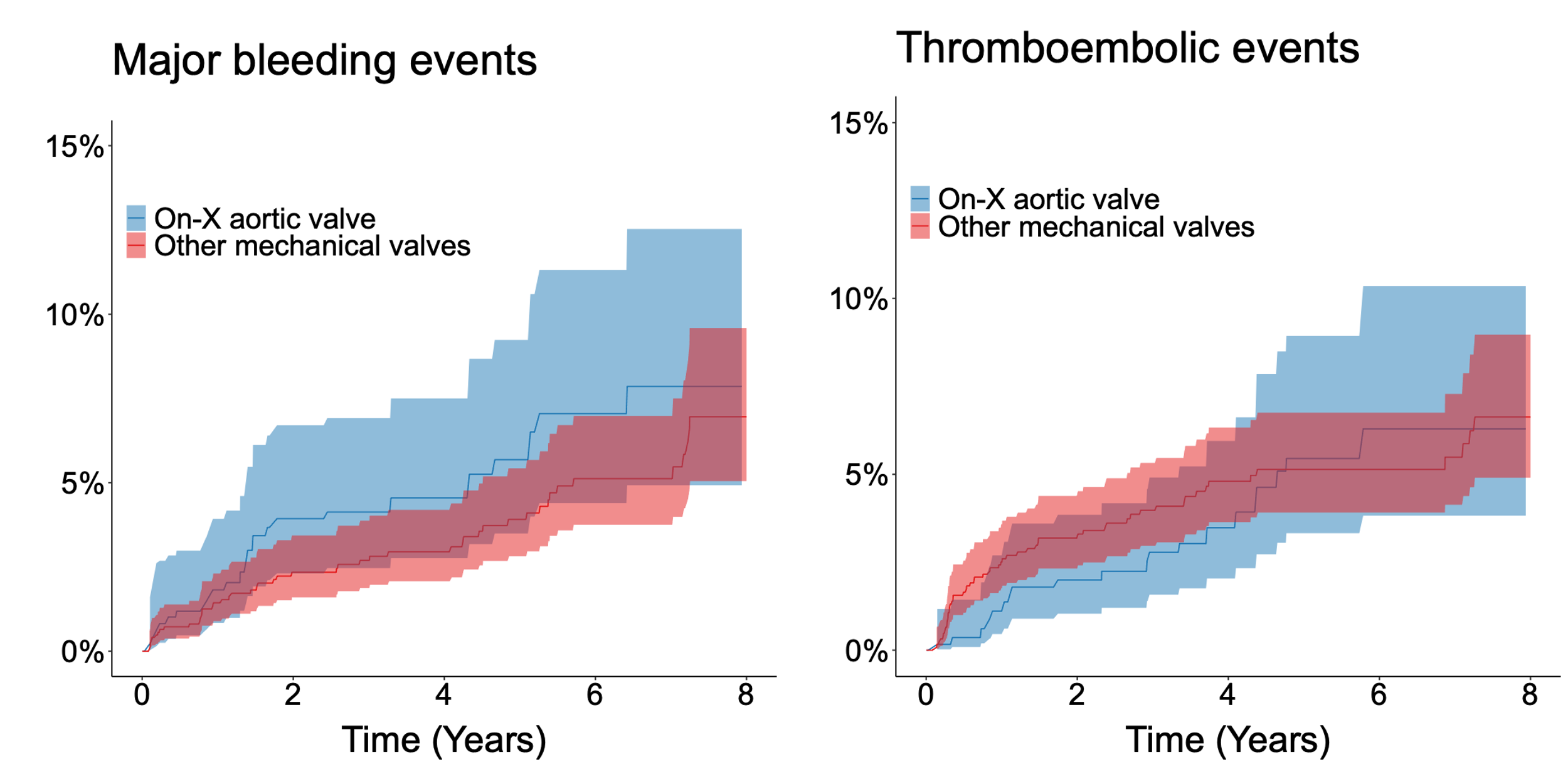


**Supplemental Figure S24:** Age distribution by valve group.


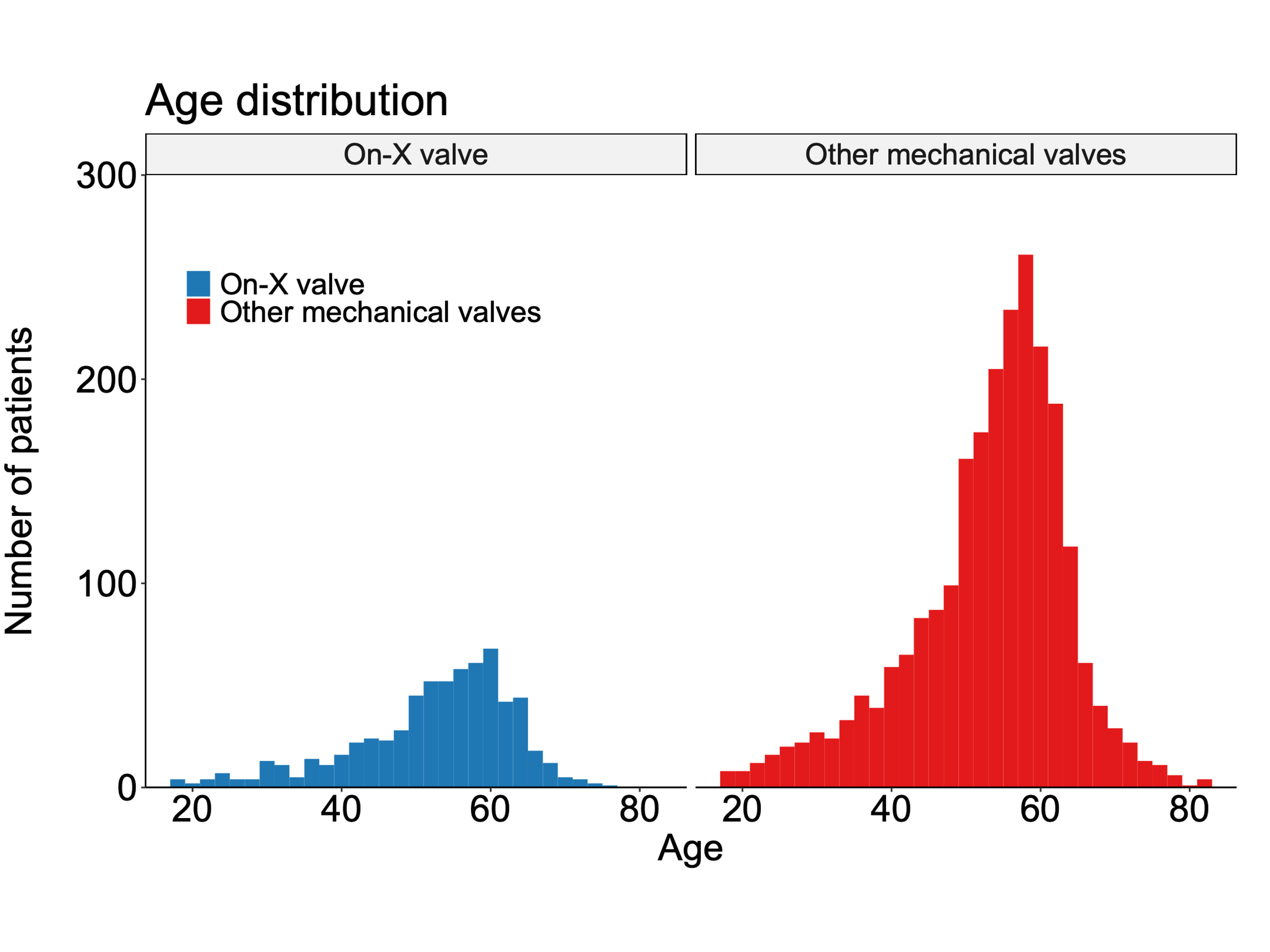

Supplement: ivaf182_Supplementary_Data [file ivaf182_supplementary_data.docx]
